# Quasi‐Periodic Porous Structures‐Based Temperature and Pressure Dual‐Mode Electronic Skin for Material Cognition

**DOI:** 10.1002/advs.202512714

**Published:** 2026-01-04

**Authors:** Xiaoguang Gao, Chengzhen Xue, Xiaoliang Zhang, Xuejuan Meng, Xiaochun Li, Li Niu

**Affiliations:** ^1^ Institute of Biomedical Precision Testing and Instrumentation College of Artificial Intelligence Taiyuan University of Technology Taiyuan China; ^2^ School of Chemical Engineering and Technology Sun Yat‐Sen University Zhuhai China; ^3^ Guangzhou Key Laboratory of Sensing Materials & Devices, Center for Advanced Analytical Science, School of Chemistry and Chemical Engineering Guangzhou University Guangzhou China

**Keywords:** material cognition, neural network, quasi‐periodic porous structure, temperature and pressure sensing

## Abstract

It is of great significance to prepare a temperature and pressure (T‐P) dual‐mode electronic skin (DMES) with a controllable porous structure and use it to achieve material cognition. In this work, a quasi‐periodic porous structure‐based T‐P DMES was proposed, exhibiting excellent performance in material cognition. The quasi‐periodic porous structure of the electronic skin was prepared through the interaction of confined two‐dimensional bubbles and polydimethylsiloxane (PDMS). After attaching graphene, PEDOT: PSS, and Bi_2_Te_3_ to the porous PDMS, the obtained electronic skin can precisely detect and distinguish T‐P stimuli without crosstalk. Benefiting from the quasi‐periodic porous structure, the temperature and pressure sensing performance of the electronic skin can be precisely constructed and optimized by changing the size of the porous structure, which is impossible for electronic skin with a random porous structure. By analyzing the thermoelectric and piezoresistive signals of the electronic skin and combining them with a convolutional neural network, the electronic skin can identify 33 different materials, including materials with similar softness or thermal conductivity, cotton fabrics with different textures, and even diverse alloys, with an accuracy of 97.64%. The proposed T‐P DMES significantly outperforms both existing electronic skins and human skin in terms of material cognition.

## Graphical abstract

A quasi‐periodic porous structure‐based temperature and pressure dual‐mode electronic skin was proposed. Benefiting from the quasi‐periodic porous structure, the temperature and pressure sensing performance of the electronic skin can be precisely constructed and optimized by changing the size of the porous structure. By analyzing the thermoelectric and piezoresistive signals of the electronic skin and combining them with a convolutional neural network, the electronic skin can identify 33 different materials, including materials with similar softness or thermal conductivity, cotton fabrics with different textures, and even diverse alloys, with an accuracy of 97.64%.

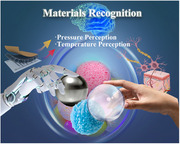

## 1 Introduction

The human skin's ability to perceive temperature and pressure (T‐P) stimuli originates from temperature‐ and touch‐sensing receptors in the skin, such as transient receptor potential (TRP) channels and Piezo proteins, which enable it to infer the material properties of external objects upon contact [1, 2]. The development of electronic skins provides a viable solution for replicating partial tactile functions of biological skin [3, 4, 5, 6, 7, 8]. Electronic skins used for material cognition can significantly improve the perception capabilities of robots and have good application prospects in smart medical care, human‐computer interaction, and other fields [9, 10, 11]. In the past few decades, a series of electrical skins based on temperature or pressure sensing have been developed and used for material cognition by mimicking the tactile perception capabilities of human skin [12, 13, 14, 15, 16, 17, 18, 19, 20, 21, 22, 23, 24, 25]. The electronic skins based on pressure sensing utilize the piezoresistive effect, capacitive effect, or piezoelectric effect to enable material cognition by detecting signal changes during contact with a target object [12, 13, 14]. These electronic skins can distinguish different kinds of materials by recognizing their softness, surface texture features. The electronic skin based on temperature sensing utilizes its thermoelectric effect to realize the identification of materials with different thermal conductivity by detecting temperature changes during contact with the target object [15, 16, 17]. Nevertheless, the unimodal characteristics of these electronic skins enable them to identify different kinds of materials by detecting only a single physical quantity, resulting in a very limited number of identifiable materials. For instance, only a very limited number of materials have distinct textures, and several others possess similar thermal conductivity and softness characteristics, leading to significant challenges in achieving material. Currently, only single‐mode electronic skins based on pressure or temperature sensing are used to achieve material recognition, and there is almost no literature on T‐P dual‐mode electronic skins (DMESs) for material recognition. Therefore, developing T‐P DMESs is of great significance for achieving material cognition.

Among the existing electronic skins based on temperature and pressure sensing, T‐P DMESs with a porous structure have attracted widespread attention due to their excellent performance, light weight, and easy integration [26, 27, 28, 29, 30, 31, 32]. In 2022, Wu et al., used freeze‐drying to prepare T‐P dual‐mode aerogels and successfully detected the characteristics of arterial pulse pressure waves under different body temperature conditions [32]. They used Ti_3_C_2_Tx MXene nanosheets as the main building block and thermal‐responsive material, along with semi‐crystalline poly (ethylene oxide) as the intercalation material, to achieve simultaneous detection and differentiation of T‐P stimuli. In 2023, Gao et al., prepared porous polydimethylsiloxane (PDMS) using a sacrificial template method [30]. More specifically, Ti_3_C_2_TxMXene was attached to the porous PDMS surface as a single active material to fabricate a high‐performance T‐P DMES. The electronic skin could simultaneously detect T‐P stimuli and convert them into independent thermoelectric voltage and relative resistance changes without crosstalk. However, the shapes and distributions of the porous structure of the T‐P DMESs developed using the above preparation methods are random, which poses severe challenges to their performance and practical application [33, 34, 35, 36, 37]. First, the random porous structure poses challenges to the controllability of both the structure and performance of the electronic skins. Afterwards, the random porous structure may lead to the concentration of stress distribution, which greatly affects the mechanical properties of the electronic skins and the conduction of electrical signals. The ordered porous structure can be prepared using 3D printing and ordered pore template methods. The 3D printing technique has been widely used in the fabrication of flexible pressure sensors with a porous structure [26, 34, 38]. The 3D printing technique has enabled exceptional control over the internal structure of pressure sensors, making it possible to construct porous microstructures within them. By integrating these microstructures into flexible sensors with various sensing mechanisms, 3D printing significantly improves performance metrics such as sensitivity, response time, and durability. However, compared with freeze drying and sacrificial template methods using sugar cubes or salt as templates, its insufficient printing efficiency and high cost still need to be addressed [39, 40, 41]. The printing efficiency and cost issues involved in the 3D printing technique still hinder its large‐scale application. The template‐based methods are capable of ensuring pore periodicity. However, the prerequisite for ensuring the periodicity of porous structures is that the structure of the template itself is periodic, which involves the design and fabrication of the template. Therefore, a large number of templates with different parameters need to be prepared to meet the different performance requirements of the electronic skins, which undoubtedly greatly increases the cost. Therefore, developing T‐P DMESs with controllable structure, simple preparation method, and excellent performance is of great significance for the material cognition.

In this study, a quasi‐periodic porous structure‐based T‐P DMES was successfully developed, exhibiting excellent performance in material cognition. During the preparation of the quasi‐periodic porous structure, the upper and lower layers of abrasive papers form a confined two‐dimensional (2D) space. The ethanol on the abrasive paper surface generates confined 2D bubbles under heating and interacts with PDMS to form a quasi‐periodic porous structure. Our proposed preparation method has certain advantages in terms of cost and preparation efficiency. In addition, our proposed preparation method is not limited by a template nor requires the extreme experimental conditions of freeze‐drying, such as low temperature and low pressure. In addition, different from the conventional electronic skins with a random porous structure, the temperature and pressure sensing performance of this electronic skin can be precisely constructed and optimized by changing the size of the porous structure. The electronic skin exhibits excellent performance in both pressure sensing (high sensitivity (50.04 kPa^−1^), low detection limit (5 Pa), and good stability (10000 cycles)) and temperature sensing (low detection limit (0.1 K) and good stability (1000 cycles)). When used for material recognition, the electronic skin, combined with a convolutional neural network (CNN) model, can accurately identify 33 different materials, which include materials with similar softness, thermal conductivity, and even different types of alloys, reaching an average recognition accuracy of 97.64%. The material cognition performance of the developed electronic skin is far superior to that of human skin and other types of electronic skin.

## 2 Results and Discussion

### 2.1 Electronic Skin Preparation and Characterization

**FIGURE 1:**
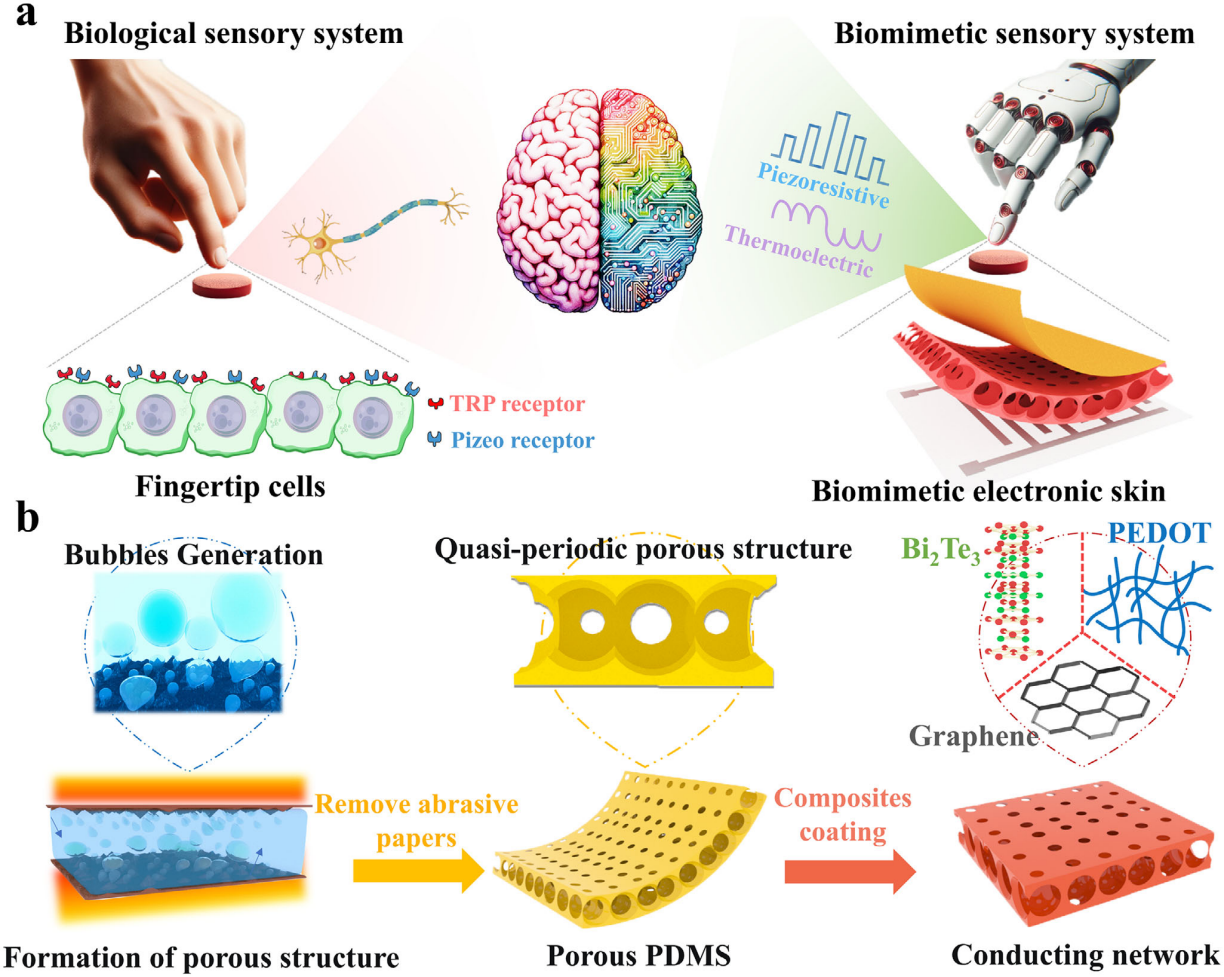
Preparation process of electronic skin. (a) Quasi‐periodic porous structure‐based electronic skin mimicking the T‐P perceptions of human skin; (b) Fabrication process of the electronic skin.

When human skin comes into contact with an object, the TRP and Piezo receptors on the cell membranes can convert external T‐P stimuli into potential changes, which are transmitted through the nervous system to the brain for further analysis. The proposed quasi‐periodic porous structure‐based T‐P DMES mimics the function of human skin, realizing the functions of both the TRP and Piezo receptors (Figure 1a). The electronic skin can respond to external T‐P stimuli. The output signals are collected and analyzed through a CNN to ultimately realize material cognition. Figure 1b illustrates the simplified preparation process of the quasi‐periodic porous structure‐based T‐P DMES. The confined 2D space formed by the upper and lower abrasive papers was filled with PDMS. An ethanol solution was added dropwise to fully wet the surface of the abrasive papers. When both the upper and lower layers of the abrasive papers were heated, a large number of bubbles were generated on the abrasive papers and entered the PDMS to form 2D bubble arrays. As PDMS solidifies, a quasi‐periodic porous structure was successfully prepared. Subsequently, the electronic skin was prepared by attaching PEDOT: PSS, Bi_2_Te_3_, and graphene on the porous PDMS to enable T‐P sensing. The detailed preparation process of the electronic skin can be found in Figure S1. According to the classical bubble theory, the formation of bubbles first involves the separation of the gas‐liquid phase [42]. The arrangement of bubbles is greatly influenced by their surrounding environment [43, 44]. In the preparation process of our proposed quasi‐periodic porous structure, ethanol solution was heated and volatilized, resulting in the separation of gas‐liquid phases and the formation of bubbles. Afterwards, during the evolution of the bubbles, they will be affected by the spinous structure of the abrasive paper surface and the confined 2D space formed by the upper and lower layers of abrasive papers to form a quasi‐periodic porous structure. Different kinds of abrasive papers. 400, 800, 1200, 1500, 2000, and 2500 were used for the preparation of a quasi‐periodic porous structure (Figure S2). It can be observed that when abrasive papers are not used. 400, 800, 1200, and 1500 were used to prepare the quasi‐periodic porous structure. The large spacing between the spinous structure on the abrasive papers results in weak confinement of the bubbles, which leads to a larger‐sized random porous structure. For abrasive paper 2500, its nearly flat surface structure makes it difficult to confine the generated bubbles, so its corresponding porous structure was randomly distributed. It can be observed that the porous structure corresponding to abrasive paper no. 2000 in Figure S2e exhibits a quasi‐periodic arrangement. In addition, the size of the porous structure was also relatively consistent. In addition, during the preparation of the quasi‐periodic porous structure, the concentration of ethanol solutions directly affects the generation rate and quantity of bubbles. Ethanol solutions with lower concentrations can only produce a smaller number of bubbles, which ultimately leads to a less porous structure. When the concentrations of the ethanol solution were too high, the rapid bubble generation process led to uneven distribution of bubbles in PDMS. Heating temperature also significantly impacts the quasi‐periodic porous structure. Lower heating temperatures result in slower ethanol evaporation, preventing the formation of a sufficiently rich porous structure. However, higher heating temperatures can cause ethanol to evaporate instantly, resulting in an oversized and unevenly distributed porous structure. Therefore, different ethanol solution concentrations and heating temperatures were used to prepare porous PDMS (Figure S3). It can be observed that the density of the porous PDMS was the lowest when the heating temperature and ethanol solution concentration were 70°C and 75 wt.%, respectively. In addition, it has been demonstrated that the height of the confined 2D space formed by two pieces of abrasive paper, i.e., the distance between them, affects the size of the pores, and thus, the density of the porous PDMS. The discussion on the effect of the distance between abrasive papers on porous PDMS density can be found in Figure S4. It has been observed that when the distance between abrasive papers exceeds 2.0 cm, the distribution of the porous structure changes from periodic to random. It can be observed that the density of porous PDMS increases with the increase of the distance between the abrasive papers, while the porosity decreases with the increase of the distance between the abrasive papers. The above preparation process is not limited by a template, and does not require extreme experimental conditions, e.g., low temperature and low pressure. Therefore, it offers advantages such as low cost and simplicity, providing a new way for large‐scale industrial manufacturing. After preparation, the porous PDMS was treated with ultraviolet (UV)/ozone to increase its hydrophilicity, which is beneficial for the attachment of nanomaterials. The changes in the contact angle of the porous PDMS before and after UV/ozone treatment prove that its hydrophilicity was significantly improved (Figure S5). Organic–inorganic hybrid PEDOT: PSS/B_i2_Te_3_ has excellent thermoelectric properties and was used for temperature sensing. The high electrical conductivity and sheet‐like structure of graphene not only facilitate efficient current transmission but also trigger significant changes in electrical resistance in response to external pressure.

**FIGURE 2:**
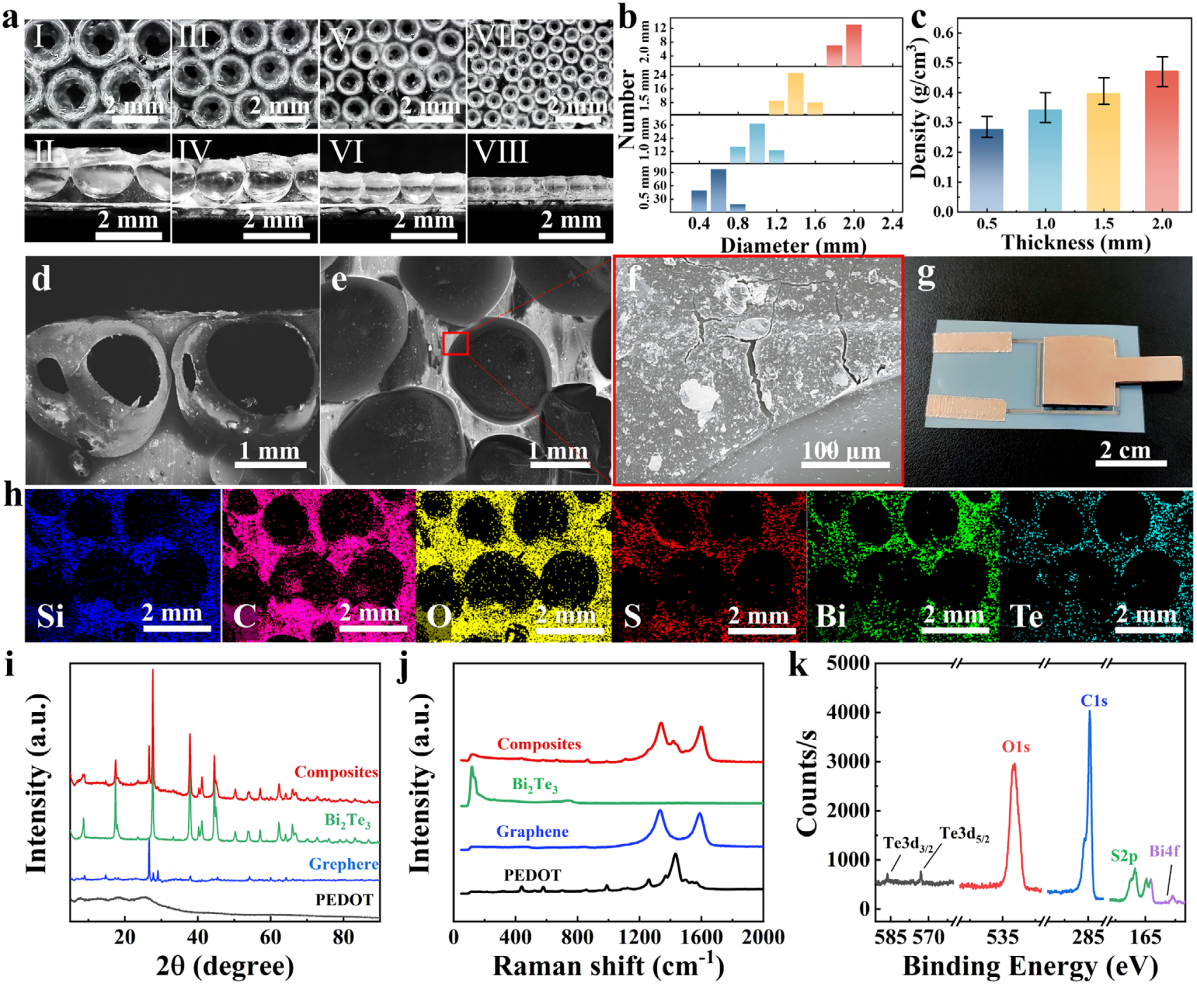
Characterization of electronic skin. (a) Photographs of the surface and cross‐section of porous PDMS prepared by two abrasive papers at different distances (2, 1.5, 1, and 0.5 mm); (b) Diameter distributions of the porous structure prepared with different distances between two abrasive papers; (c) Effect of different distances of abrasive papers on the density of porous PDMS. (d–f) SEM images of the cross‐section and surface of porous PDMS with attached graphene, PEDOT: PSS, and Bi_2_Te_3_, respectively; (g) Photographs of the electronic skin; (h) EDS mapping images of the porous PDMS with attached composites; (i) Raman spectra of graphene, PEDOT: PSS, Bi_2_Te_3_, and composites; (j) XRD patterns of graphene, PEDOT: PSS, Bi_2_Te_3_, and composites; (k) XPS spectrum of composites on the porous PDMS.

The photographs of the surface and cross‐section of porous PDMS prepared by the upper and lower abrasive papers at different distances (2, 1.5, 1.0, and 0.5 mm) are shown in Figure 2a. It was found that the pores of the PDMS were spherical in shape. Moreover, these spherical pores were arranged quasi‐periodically, which is attributed to the interaction between the confined 2D bubble array and PDMS. The diameter distribution of the porous structure corresponding to different distances between abrasive papers is shown in Figure 2b. It can be observed that the diameters of the porous structure increase with the increase of the distance between the abrasive papers and are basically consistent with the distance between the abrasive papers. The densities of porous PDMS corresponding to different distances between abrasive papers are shown in Figure 2c. It can be observed that the density of porous PDMS decreases as the distance between abrasive papers decreases, which is caused by the different sizes of the quasi‐periodically arranged porous structure. It can be concluded that the difference in the size of the porous structure leads to the difference in the density of the porous PDMS, which provides a way to dynamically regulate the performance of the electronic skin. Micro‐CT technique (ZEISS Xradia 620 Versa) was used to characterize PDMS with a quasi‐periodic porous structure. The CT images of porous PDMS prepared with different distances of abrasive papers are displayed in Figure S6. It can be observed that the porous structure presents a quasi‐periodic arrangement. Scanning electron microscopy (SEM) images of the cross‐section and surface of the composite porous PDMS are shown in Figure 2d, e. It can be observed that the attached nanomaterials altered the brightness of the porous PDMS. In the magnified SEM image (Figure 2f), the surface of the porous PDMS with attached nanomaterials can be observed. A photograph of the encapsulated electronic skin is presented in Figure 2g. The quasi‐periodic porous structure‐based electronic skin was manufactured using a typical packaging method, where interdigitated electrodes and conductive copper foil are attached to the two surfaces of the sensor using silver paste. The electrodes on the left were used to collect the piezoresistive signal of the electronic skin. A source meter (Keithley, 2600B) was used to measure the changes in relative resistance of the electronic skin when different pressures were applied. The electrode on the right was used to apply a temperature difference and serves as one of the electrodes for thermoelectric voltage output. Hence, the resistance change induced by pressure and the thermoelectric voltage generated by temperature differences can be measured simultaneously without crosstalk. The elemental mapping images revealed a homogeneous distribution of C, S, Bi, and Te over the entire porous PDMS, confirming the homogeneous deposition of graphene, PEDOT: PSS, and Bi_2_Te_3_ (Figure 2h). The Si and O elements were derived from the porous PDMS. Raman spectroscopy was used to characterize the PEDOT: PSS, Bi_2_Te_3_, graphene, and composites on the PDMS surface. It can be observed that the G band (1588 cm^−1^), i.e., a characteristic peak of graphene, was shifted to 1596 cm^−1^ in the composites, which was caused by the strong π–π interaction between PEDOT: PSS and graphene (Figure 2i). Furthermore, all Raman peaks corresponding to graphene, Bi_2_Te_3_, and PEDOT: PSS were found in the Raman spectrum of the composites on the porous PDMS. XRD patterns (Figure 2j) and XPS characterization (Figure 2k) also confirmed that graphene, Bi_2_Te_3_, and PEDOT: PSS were successfully incorporated onto the porous PDMS surface.

### 2.2 Electronic Skin Performance

**FIGURE 3:**
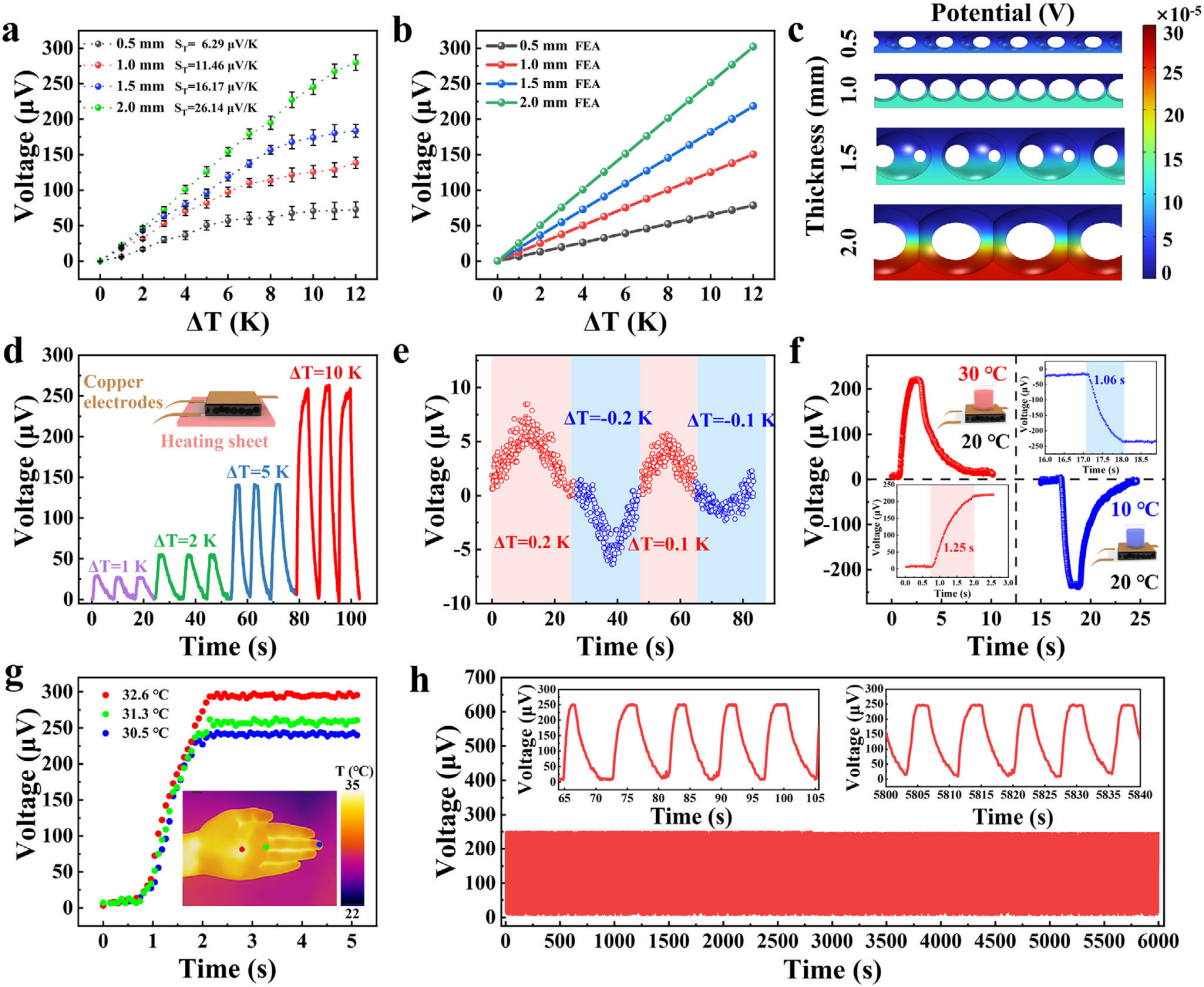
Thermoelectric properties of the electronic skin. (a, b) Experimental and simulated thermoelectric voltage as a function of the temperature gradient corresponding to porous structure of different sizes; (c) Simulated temperature and potential distributions under temperature gradient of 10 K; (d) Voltage response to different temperature gradients; (e) Voltage outputs at subtle temperature gradients (0.1 and 0.2 K); (f) Real‐time voltage response under high and low temperature stimulation; (g) Voltage responses when different regions of the palm touch the electronic skin. Infrared images show the temperature distribution of the palm; h Voltage output for 750 cycles within a temperature gradient range of 0–10 K.

The T‐P sensing properties and signal decoupling capability of the DMES have been explored. According to the Seebeck effect, a thermoelectric voltage is generated when a temperature difference exists at both ends of the electronic skin. When the electronic skin is used for temperature sensing, the Seebeck coefficient (S_T_), defined as S_T_ = ΔV/ΔT, is used to evaluate its sensitivity, where ΔV represents the thermoelectric voltage and ΔT the temperature gradient. The thermoelectric properties and temperature sensing performance of the electronic skin were assessed by measuring the voltage generated under different applied temperature gradients. PEDOT: PSS and Bi_2_Te_3_ have been used for temperature sensing due to their excellent thermoelectric properties. Figure 3a exhibits the thermoelectric voltage generated by the electronic skin under different temperature gradients corresponding to porous structures of different sizes. The positive S_T_ value results from the p‐type nature of Bi_2_Te_3_ and PEDOT: PSS, where holes serve as the charge carriers. The optimization of the mass ratio of PEDOT: PSS and Bi_2_Te_3_ can be found in Figure S7. It has been demonstrated that the S_T_ value of the electronic skin reaches its maximum when the mass ratio of PEDOT to Bi_2_Te_3_ is 2:1. It can be observed that a larger diameter of the porous structure will lead to a larger S_T_ of the T‐P DMES, which is due to the fact that more thermoelectric materials were attached to the porous structure with a larger diameter (Figure 3a). The maximum value of S_T_ reached 26.14 µV/K. Finite Element Analysis (FEA) was also used to analyze the thermoelectric properties of the electronic skin. The output thermoelectric voltages caused by different temperature gradient stimuli were simulated in Figure 3b. It can be observed that the results obtained by finite element simulation are highly consistent with the experimental results, verifying that the electronic skin realizes temperature sensing through the thermoelectric effect. In Figure 3c, a finite element simulation was used to quantitatively analyze the potential distribution of T‐P DMES corresponding to porous structures of different sizes under the same temperature gradient (10 K). It can be observed that under the stimulation of the same temperature difference, the potential distribution of the electronic skin corresponding to porous structures of different sizes has obvious differences. Figure 3d illustrates the output voltage of the electronic skin under multi‐cycle stimulation with different temperature gradients (1, 2, 5, and 10 K). It can be observed that the electronic skin could not only accurately distinguish these temperature gradients, but also its signal was highly consistent under stimulation with the same temperature gradient. According to Figure 3e, the electronic skin was able to distinguish subtle temperature gradients (0.1 and 0.2 K) with an ultra‐high signal‐to‐noise ratio, which is of great significance for realizing material cognition. In Figure 3e, the initial temperature was 20°C. Figure 3f displays the response time of the electronic skin during temperature sensing. It can be observed that when the skin was touched a hot beaker (30°C) and a cold beaker (10°C), the response times were 1.25 and 1.06 s, respectively. Fast temperature response is important for further signal processing and rapid material identification. As depicted in Figure 3g, the electronic skin was attached to different locations on the palm to distinguish tiny temperature gradient changes. It can be clearly seen that the thermoelectric voltages generated by different temperatures in the palm differed. The long‐term stability of the electronic skin for temperature sensing is exhibited in Figure 3h. Over the course of 750 cycles, the change in thermoelectric voltage caused by a temperature gradient of 10 K did not exceed 5%, proving that the electronic skin has excellent stability during temperature sensing. Such a large number of cycles also exceeds most existing literature [30, 45, 46]. In Figure 3f, it can be observed that the recovery time is significantly longer than the response time. The reason for the above phenomenon is that the hot beaker (30°C) and the cold beaker (10°C) were placed on the surface of the electronic skin to apply a temperature difference. Such direct contact will cause the temperature difference to form very quickly. When the hot beaker (30°C) and the cold beaker (10°C) were removed from the electronic skin, the temperature difference on the surface of the electronic skin would disappear only after heat conduction with the air. This is also the reason that the recovery time is significantly longer than the response time. In Figure 3h, when metal sheets were used for heat dissipation, there was no significant difference in the response and recovery times of the electronic skin.

**FIGURE 4:**
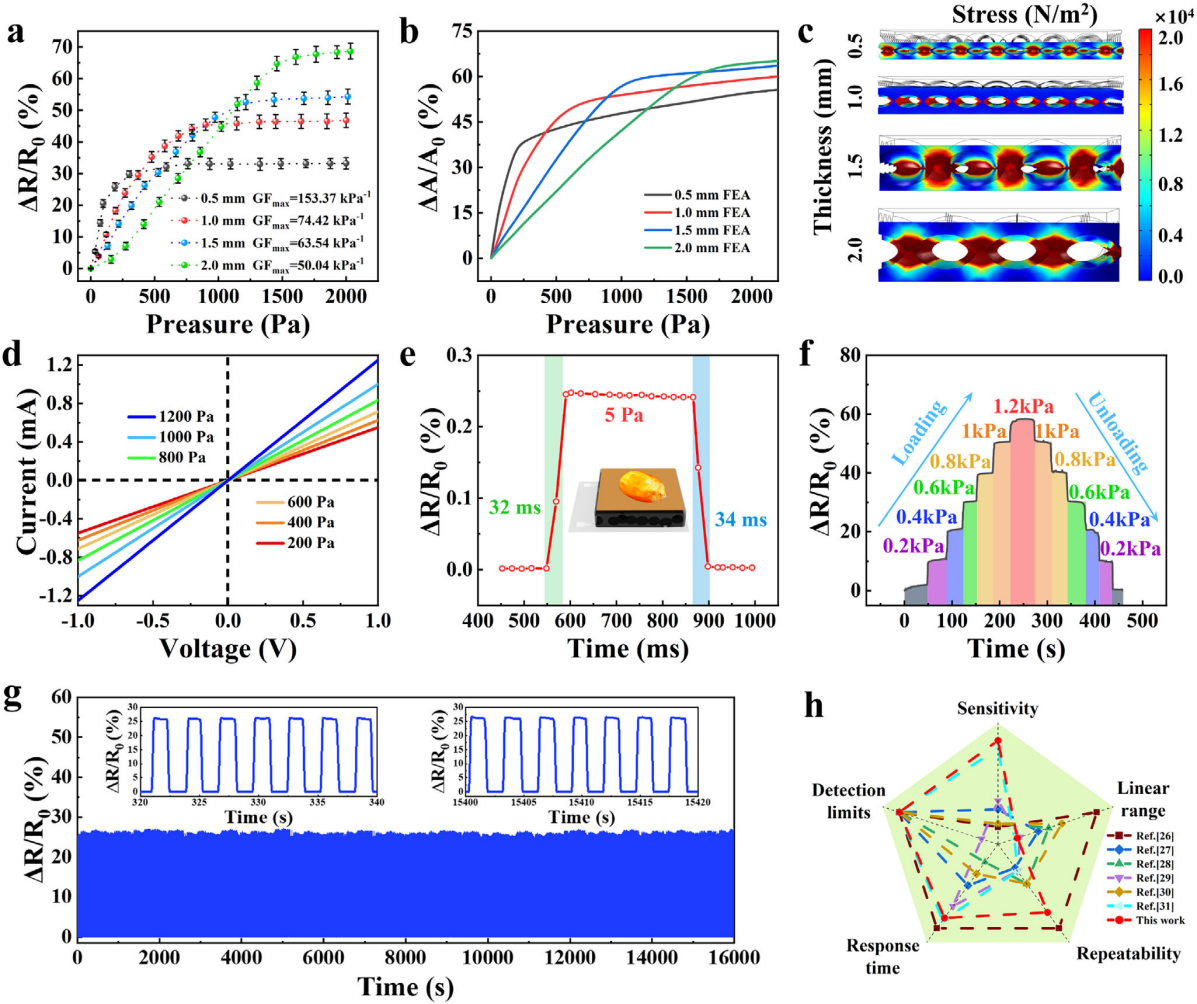
Piezoresistive properties of the electronic skin. (a) Changes in relative resistance as a function of pressure corresponding to porous structure of different sizes; (b) Simulated change in relative contact area as a function of the pressure corresponding to porous structure of different sizes; (c) Simulated pressure distributions under applied pressure of 1000 Pa; (d) The relation of the current–voltage curves of the electronic skin under various pressures; (e) Changes in relative resistance under subtle pressure variations; (f) Changes in relative resistance under different pressures; (g) Long‐term stability for pressure sensing; (h) Comparison of the performance of the proposed electronic skin and that of other sensors reported in the literatures.

The pressure‐sensing performance of the electronic skin was assessed in terms of sensitivity, response time, and stability. The gauge factor (GF) is used to characterize the pressure‐sensing sensitivity and is defined as GF = (ΔR/R_0_)/ΔP, where ΔR, R_0_, and ΔP represent the change in resistance due to pressure loading, initial resistance, and pressure change, respectively. It has already been mentioned that PEDOT: PSS and Bi_2_Te_3_ are used for temperature sensing due to their thermoelectric properties, while graphene is used for pressure sensing due to its sheet‐like structure and high conductivity. The optimization of the mass ratio of PEDOT: PSS, Bi_2_Te_3_, and graphene is discussed in Figure S7. It was found that the electronic skin has the largest GF value when the mass ratio of PEDOT: PSS, Bi_2_Te_3_, and graphene is 20:10:1. Figure 4a exhibits the relative resistance change generated by the electronic skin under different pressures corresponding to porous structures of different sizes. It was found that the porous structure with a larger diameter will lead to a smaller GF value and a wider detection range of the electronic skin. Therefore, dynamic regulation of the piezoresistive properties of the electronic skin can be achieved by adjusting the size of the porous structure. It can be observed that when the distance between the abrasive papers is 0.5 mm, the GF value of the electronic skin is the largest (153.37 kPa^−1^). It can be observed that when the distance between the abrasive papers is 2.0 mm, the electronic skin has the largest linear detection range (0–1.3 kPa). The error bars in Figure 4a represent the variability derived from three devices prepared under identical experimental conditions. Finite element analysis was utilized to analyze the piezoresistive properties of the electronic skin under different pressures. A three‐dimensional quasi‐periodic porous structure was developed and quantitatively analyzed (Figure 4b). It can be observed that the results obtained by finite element simulation are highly consistent with the experimental results. In Figure 4c, a finite element simulation was used to quantitatively analyze the pressure distribution of T‐P DMES corresponding to porous structures of different sizes under the same pressure (1000 Pa). It can be observed that under the same applied pressure, porous structure of different sizes has significantly different deformation degrees. The electronic skin with a smaller porous structure has the most obvious deformation. Nonetheless, the shape and distribution of the porous structure obtained through the template and freeze‐drying methods are random, making it difficult to quantitatively analyze its performance via modeling and simulation. Considering that a large detection range is required when the electronic skin is used to identify materials of different softness, the piezoresistive properties of the electronic skin with a porous structure with a diameter of 2 mm will be further explored. The current‐voltage curves in Figure 4d show the linear current response against voltage under different pressures, which also proves that the resistance change of the electronic skin comes from the applied pressure. Figure 4e presents the detection limit of the electronic skin during pressure sensing. The sampling frequency of the data was about 50 Hz. It can be observed that the electronic skin was able to detect a subtle pressure of as low as 5 Pa. Furthermore, the response and recovery times of the electronic skin during the placement and removal of a corn kernel were 32 and 34 ms, respectively. Figure 4f displays the changes in relative resistance of the electronic skin in response to subtle variations in applied pressure. The electronic skin was able to distinguish these subtle pressure changes, which is significant for the identification of materials with similar softness. Figure 4g presents the long‐term stability of the electronic skin in pressure‐sensing applications. Over the course of 5600 cycles, the maximum and minimum values ​​of the relative resistance change varied by no more than 5%, demonstrating its excellent long‐term stability. Such a large number of cycles also exceeds most existing literature [34, 35, 47]. To obtain a more comprehensive understanding of the pressure sensing performance of our proposed electronic skin, we compared its performance with that of other electronic skins reported in the literature (Figure 4h). We believe that only performance comparisons of the same type of electronic skin are objective and reasonable. Therefore, various T‐P DMES were used for comparison with the electronic skin we proposed. In addition, to make the performance comparison objective and reasonable, the preparation methods of electronic skins we selected from the literature covered freeze‐drying, template method, and 3D printing technique. Compared with the T‐P DMES in the literature, our proposed electronic skin has a significant advantage in sensitivity when used for pressure sensing. The electronic skin we proposed is comparable to those reported in the literature in terms of detection limit, response time, and repeatability. To demonstrate its practicality, the electronic skin was applied to detect both subtle and intense human motions (Figure S8). The electronic skin was capable of detecting both subtle human motions, such as pulse and blinking, and intense human motions, such as wrist bending, finger bending, arm bending, and knee bending. To demonstrate the advantages of electronic skin based on a quasi‐periodic porous structure in pressure sensing, we fabricated electronic skin using a template method and compared it with electronic skin based on a quasi‐periodic porous structure. The electronic skin based on a random porous structure was prepared using a sugar cube as a template. In addition, the mass ratios of Bi_2_Te_3_, PEDOT: PSS, and graphene used in the two kinds of electronic skins are the same. It can be observed that the electronic skin we proposed has a significant advantage in terms of sensitivity (Figure S9a, b). The real‐time response of the electronic skins, along with the signal corresponding to each pixel during the movement of the weight, was recorded. It can be observed that the signal of the electronic skin based on the quasi‐periodic porous structure is basically consistent during the movement of the weight, while the signal of the electronic skin based on the random porous structure fluctuates significantly (Figure S9c, d). Therefore, the electronic skin based on a quasi‐periodic porous structure exhibits significant advantages in pressure sensing.

**FIGURE 5:**
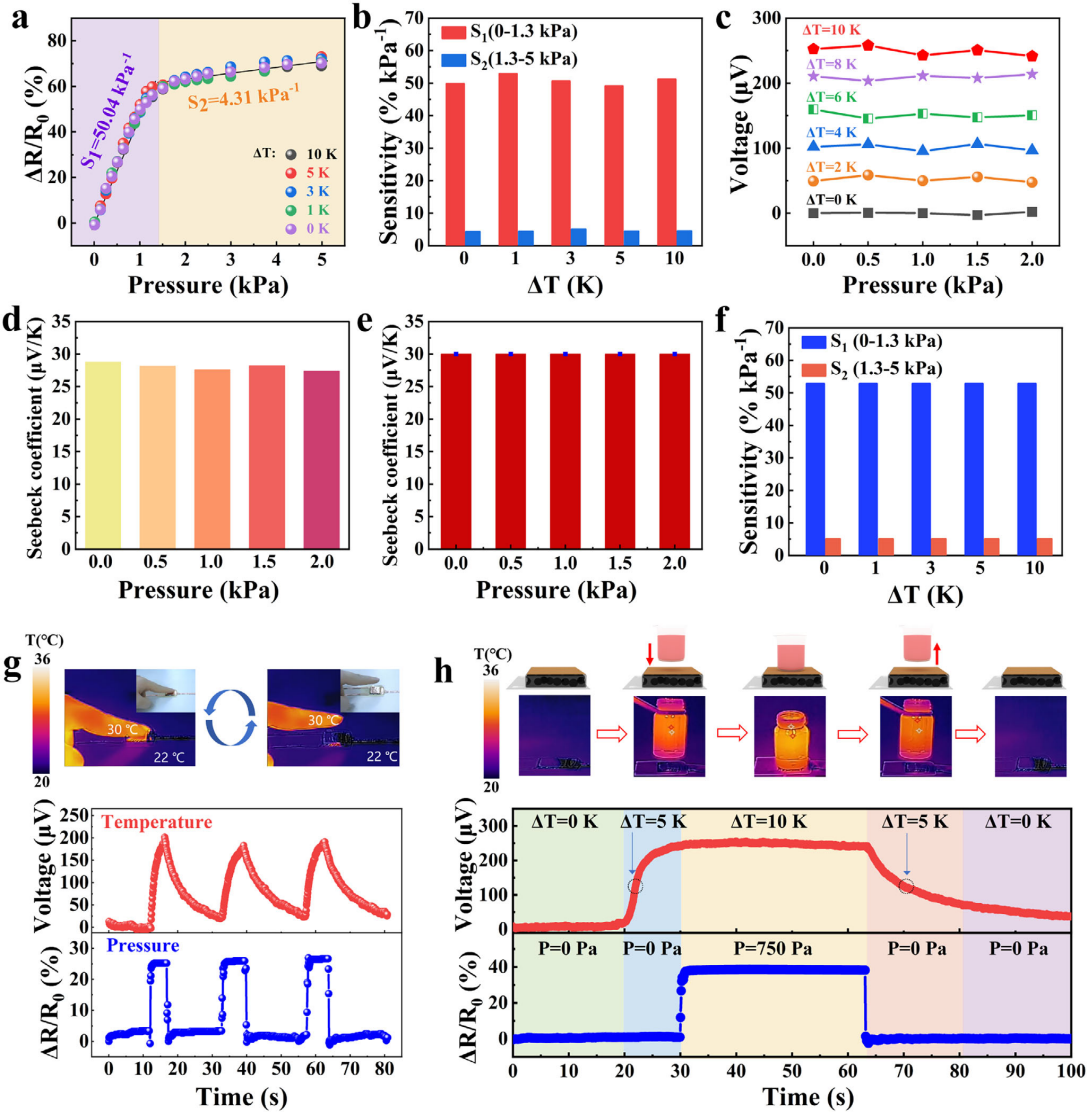
T‐P dual‐mode sensing capabilities of the electronic skin. (a) Changes in relative resistance as a function of different pressures under different ΔT; (b) GF values under different ΔT; (c) Output voltage as a function of pressure under different ΔT; (d) S_T_ under different pressures; (e, f) Simulation results for the S_T_ and GF values under different pressures and ΔT; (g) Response of the electronic skin during finger pressing; (h) Real‐time signal responses induced by a beaker of hot water.

The decoupling performance of the proposed quasi‐periodic porous structure‐based electronic skin was assessed. Figure 5a exhibits the changes in relative resistance as a function of different pressures under different temperature gradients. The GF values for the electronic skin are depicted in Figure 5b. It can be observed that temperature had no effect on relative resistance change and GF values. Figure 5c shows the thermoelectric voltage as a function of temperature gradients under different pressures. Notably, the output thermoelectric voltage was not affected by pressure changes. In addition, the S_T_ of the electronic skin appeared to be independent of the applied pressure (Figure 5d). These experimental results indicate that the electronic skin has an excellent signal decoupling capability and can accurately detect T‐P stimuli simultaneously without crosstalk. Figure 5e, f present the finite element simulation results used to analyze the response of the electronic skin when it is simultaneously stimulation by a temperature gradient and applied pressure. It can be observed that the output thermoelectric voltage remained stable under a constant temperature gradient and was unaffected by changes in pressure. At the same time, the temperature gradient had no effect on the piezoresistive properties of the electronic skin. The decoupling capability of the electronic skin is caused by the following factors. The temperature sensing performance of the electronic skin comes from the thermoelectric properties of PEDOT: PSS and Bi_2_Te_3_, which are not affected by the loaded pressures. The pressure‐sensing performance of this electronic skin comes from the changes in the conductive network under different applied pressures, which is also independent of external temperature changes. In addition, the thermoelectric voltages and relative resistance changes of the electronic skin can be output completely independently and simultaneously. Subsequently, different application scenarios were utilized to verify the decoupling capability of the electronic skin. Figure 5g illustrates the response of the electronic skin to finger pressure. The infrared photographs reveal the temperature difference between the finger and the electronic skin as well as the pressing process. In addition, Figure 5g presents the thermoelectric voltage generated by the temperature difference and the changes in relative resistance induced by the pressing process. The infrared images in Figure 5h display a beaker of hot water being placed on, and then moved away from the electronic skin. A significant thermoelectric voltage was generated when the beaker gradually approached the electronic skin. It can be observed that the thermoelectric voltages of the electronic skin were 121 and 256 µV when the temperature difference was 5 and 10 K, respectively. Such thermoelectric voltage values are consistent with the results in Figure 3a. However, the relative resistance changed only when the electronic skin came into contact with the beaker. It can also be observed that the pressure was removed, and the relative resistance change of the electronic skin also decreases to zero. Results on the interaction between the electronic skin and beakers containing water at different temperatures are provided in Figure S10. The above experimental results prove that the electronic skin can synchronously perceive T‐P stimuli without crosstalk.

### 2.3 Deep Learning‐Based Material Cognition

**FIGURE 6:**
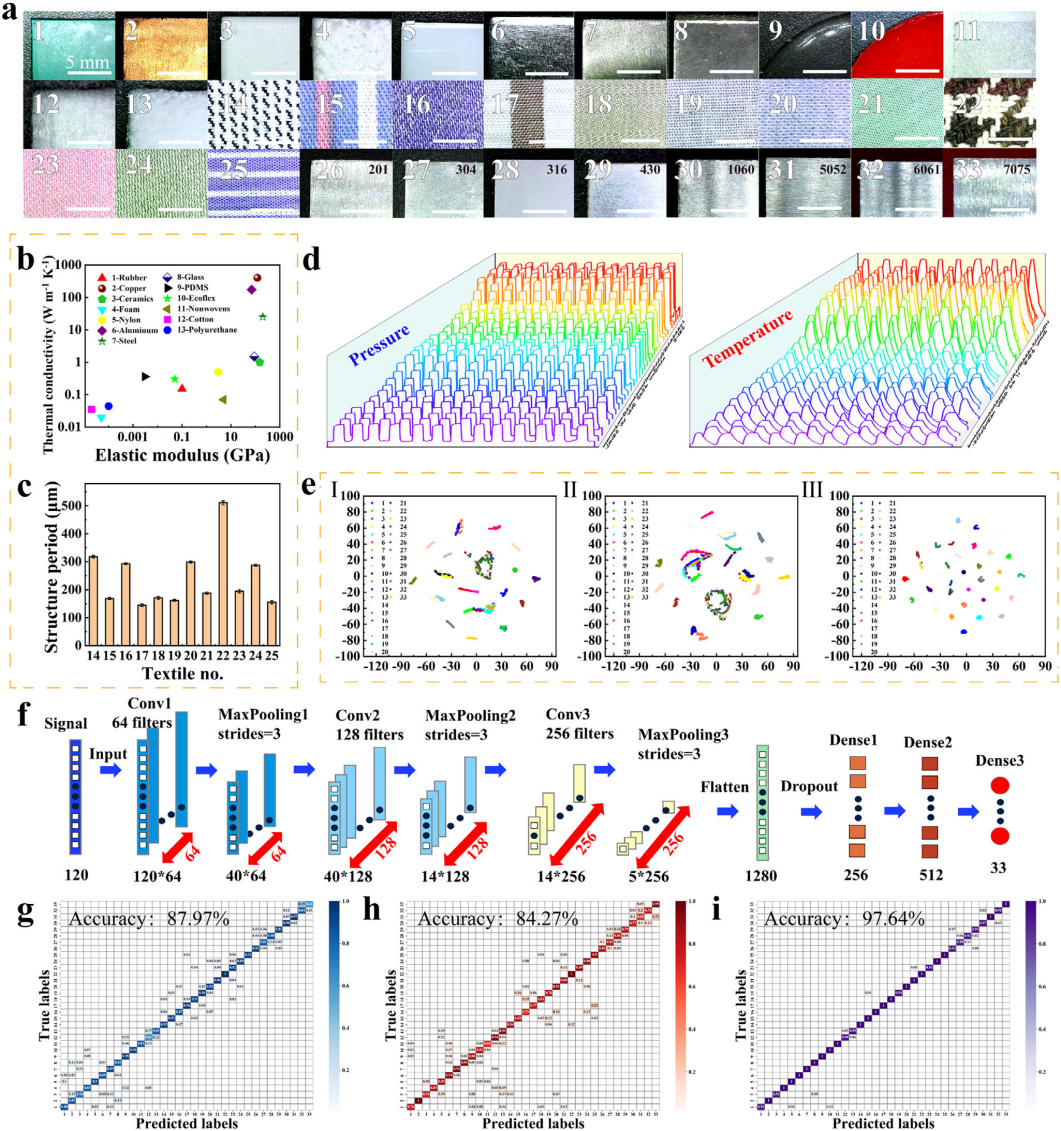
Recognition of materials using the electronic skin. (a) Images of 33 materials selected for material cognition; (b) 13 materials with similar softness or thermal conductivity (No. 1–13); (c) Structure periods of 12 textiles (No. 14–25); (d) Relative resistance changes and thermoelectric voltages generated when pressing different materials with the electronic skin; (e) t‐SNE visualization of the different datasets collected from the 33 selected materials; (f) Detailed structure of the CNN model; (g–i) Confusion matrices for material cognition using the relative resistance change dataset, thermoelectric voltage dataset, and combined dataset.

To realize material cognition, the human skin responds to T‐P stimuli through TRP and Piezo receptors in cells. Herein, the quasi‐periodic porous structure‐based T‐P DMES was combined with a CNN to achieve accurate recognition of different materials. For electronic skin based on temperature sensing, the heat transfer process when the electronic skin contacts the target material will cause the output thermoelectric voltage of the electronic skin to change, thereby realizing material recognition. Therefore, the thermal conductivity of the target material can greatly affect the output voltage of the electronic skin. For electronic skin based on the piezoresistive effect, its resistance will change differently when the electronic skin comes into contact with materials of different softness, thereby achieving material recognition. Therefore, the Young's modulus of the target material can greatly affect the relative resistance change of the electronic skin. Figure 6a depicts images of the 33 selected materials used for material cognition. The surface of materials No. 1–13 was smooth. These materials possessed similar softness, e.g., copper, aluminum, steel, glass, and ceramics, or similar thermal conductivity, e.g., PDMS, Ecoflex, rubber, nylon, and ceramics. The thermal conductivity and Young's moduli of the above 13 materials are presented in Figure 6b. Clearly, distinguishing the above materials by relying solely on the tactile perception of human skin is impossible. Materials No. 14–25 were cotton fabrics with different surface textures. The structural period of the above cotton textiles is exhibited in Figure 6c. It can be observed that the structural periods of the textures of most cotton fabrics were identical, making it impossible to distinguish these cotton fabrics by relying solely on human tactile perception. The remaining materials (No. 26–33) were different types of alloys. Overall, the study included materials with similar softness or thermal conductivity, materials with different surface textures, and diverse alloys, exceeding the range of materials examined in previous studies. The process of using the electronic skin to identify 33 materials was recorded in Movie S1. The electronic skin was first fixed to the surface of the manipulator. In the material cognition process, the electronic skin was translated a specific distance (0.5 mm) after making contact with the target material. In addition, the electronic skin was heated to 35°C before contacting the selected materials. The relative resistance changes and thermoelectric voltage of the electronic skin were then collected by a source meter. Finally, the collected signals were processed by the CNN model to output relevant results of material cognition. The changes in relative resistance and the thermoelectric voltage during the interactions between the electronic skin and the different materials are displayed in Figure 6d. It was observed that variations in the softness of the selected materials induced distinct changes in relative resistance. Differences in thermal conductivity also led to district temperature differences between the upper and lower surfaces of the electronic skin, generating different levels of thermoelectric voltage. The relative resistance changes and thermoelectric voltage signals of the electronic skin to materials with similar softness (Movie S2), thermal conductivity (Movie S3), and textures (Movie S4) are recorded in real time. The robotic arm and the display screen were filmed in the same shot. The changes in relative resistance and the thermoelectric voltage of the 33 materials were used to establish three datasets. These datasets included data regarding (1) changes in relative resistance, (2) changes in thermoelectric voltage, and (3) combined changes in relative resistance and thermoelectric voltage of the selected materials. The number of relative resistance changes and thermoelectric voltages corresponding to each material is 100. To better understand the inherent structure of these datasets, t‐distributed stochastic neighbor embedding (t‐SNE) was utilized to project higher‐dimensional data into a 2D space and enable data visualization. Figure 6e shows the distinction cluster of the different datasets collected for 33 materials. It can be observed that the combined dataset of relative resistance changes and thermoelectric voltages could be effectively visualized and distinguished in the 2D space.

CNNs are known for their excellent feature extraction capabilities and are widely used in the field of material cognition. A total of 6600 data points (half of relative resistance changes and half of thermoelectric voltages) were collected and allocated to the training and test sets at a ratio of 7:3. To build a CNN model, optimization of parameters such as the number of convolution layers, size of the convolution core, and depth of the full connection layer are critical to model performance. The specific configuration and parameters of the model are shown in Figure 6f. It can be observed that when the dataset consisted only of relative resistance changes or thermoelectric voltages, the accuracy rates were 87.97% and 84.27%, respectively, demonstrating the limitations of SMESs. However, when the combined dataset was used, the accuracy rate reached 97.64%. In addition, the electronic skin was also combined with other deep learning models, such as Lenet‐5, VGG16, ResNet, and AlexNet, for material recognition. It has been demonstrated that the CNN model has obvious advantages in accuracy and loss value compared to other deep learning models (Figure S11). Moreover, the electronic skin was combined with a CNN model to identify materials with similar softness or thermal conductivity (Figure S12), cotton materials with different textures (Figure S13), and diverse alloys (Figure S14). The above experimental results indicate that the T‐P sensing capabilities endow the electronic skin with apparent advantages in material cognition. The performance of the proposed electronic skin and other skins used for material cognition is compared in Table S1. The proposed quasi‐periodic porous structure‐based T‐P DMES not only surpasses the material cognition performance of existing electronic skins but also significantly outperforms human skin.

## 3 Conclusion

The proposed quasi‐periodic porous structure‐based T‐P DMES exhibited superior performance in material cognition, with the key highlights listed below: (1) The shape and diameter of the prepared quasi‐periodic porous structure are controllable, which is critical for quantitatively analyzing the relationship between the performance of the electronic skin and its porous structure; (2) The proposed electronic skin can accurately identify materials with similar softness and thermal conductivity, cotton fabrics with different textures, and even diverse alloys. It exhibits tactile perception capabilities far superior to human skin and other electronic skins in terms of material cognition; (3) The quasi‐periodic porous structure can be successfully synthesized through the interaction between a 2D bubble array in a confined space and PDMS.

In summary, a quasi‐periodic porous structure‐based T‐P DMES was successfully prepared and used for material cognition. The porous PDMS was prepared through the interaction of confined 2D bubbles and PDMS. Graphene, PEDOT: PSS, and Bi_2_Te_3_ enabled the electronic skin to respond to T‐P stimuli without crosstalk. The electronic skin exhibited excellent performance when used for T‐P sensing. It has been demonstrated that, combined with deep learning, the electronic skin can identify materials with similar softness or thermal conductivity, cotton fabrics with different textures, and even diverse alloys. Overall, the excellent tactile perception demonstrates that the electronic skin not only has potential applications in robotics but is also expected to be applied in healthcare and consumer electronics, enhancing tactile functions for humans.

## 4 Experimental Section/Methods

### 4.1 Materials

Graphene powder was purchased from Nanjing Xianfeng Nanotechnology Co., Ltd, PEDOT: PSS was purchased from Xi'an Qiyue Biotechnology Co., Ltd, Bi_2_Te_3_ was purchased from Shanghai MacLean Biochemical Technology Co., Ltd, and PDMS was purchased from Dow Corning. The packaging material of the T‐P DMES was polyurethane film. The interdigitated electrodes used in the experiments were manufactured using a flexible microelectronic printer (Prtronic, Scientific 3).

### 4.2 Characterization

The surface morphology of the electronic skin was characterized via scanning electron microscopy (SEM; HITACHI, SU8010) and energy dispersive spectrometry (EDS). Raman spectroscopy (Renishaw, Qontor), X‐ray diffractometry (XRD; Malvern Panalytical, Aeris), and X‐ray photoelectron spectrometry (XPS; Thermo Scientific ESCALAB Xi+) were used to characterize the nanomaterials used in the experiments. Lastly, a densitometer (Xiamen Yishite ST‐300C) was used to measure the density of the porous PDMS.

### 4.3 Preparation of PDMS with the Quasi‐Periodic Porous Structure

In the porous PDMS preparation process, two abrasive papers (2 cm × 2 cm) formed a confined space. The distance between the upper and lower abrasive papers was 2 mm. 100 µL of 75 wt. % ethanol solution was added to completely wet the abrasive paper surface. Subsequently, the space between the abrasive papers was filled with PDMS and heated (70°C, 1 h) to cure. The preparation of porous PDMS was completed after removing the upper and lower abrasive papers.

### 4.4 Preparation of Electronic Skin

The porous PDMS was treated with a UV/ozone cleaner to improve its hydrophilicity. PEDOT: PSS/Bi_2_Te_3_/graphene at different mass ratios were sonicated for 30 min to form homogeneous suspensions. The porous PDMS was immersed in the as‐prepared PEDOT: PSS/Bi_2_Te_3_/graphene suspensions for 40 min under mild sonication. Then, it was dried at 70°C for 1 h. After completing the interdigital electrode fixation and packaging processes, the electronic skin was successfully prepared.

### 4.5 Performance Measurements

The pressure and temperature sensing properties of the electronic skin were characterized. A universal testing machine (Shenzhen Suns CMT6502) was used to apply different pressures to the electronic skin. A source meter (Keithley, 2600B) was used to measure the changes in relative resistance of the electronic skin when different pressures were applied. Two Peltier elements were used to generate temperature gradients, and an infrared thermal camera (HIKMICRO, K20) was used to record the temperature distribution on the electronic skin surface. The voltage generated by the temperature difference was recorded and output by the source meter (Keithley, 2600B).

## Conflicts of Interest

The authors declare no conflict of interest.

## Supporting information




**Supporting File 1**: advs73682‐sup‐0001‐SuppMat.docx.

**Supporting File 2**: advs73682‐sup‐0002‐MovieS1.docx.

**Supporting File 3**: advs73682‐sup‐0003‐MovieS2.docx.

**Supporting File 4**: advs73682‐sup‐0003‐MovieS3.docx.

**Supporting File 5**: advs73682‐sup‐0004‐MovieS4.docx.

## Data Availability

The data that support the findings of this study are available from the corresponding author upon reasonable request.
