# Supplementary material for: Quasi‐Periodic Porous Structures‐Based Temperature and Pressure Dual‐Mode Electronic Skin for Material Cognition
Source: Adv Sci (Weinh). 2026 Jan 4;13(16):e12714. doi: 10.1002/advs.202512714 (PMC13042795; doi:10.1002/advs.202512714)
Supplement: Supplementary file 1 — Supporting File 1: advs73682‐sup‐0001‐SuppMat.docx. [file ADVS-13-e12714-s001.docx]

Supporting Information

**Quasi-periodic porous structures-based temperature and pressure dual-mode electronic skin for material cognition**

***Xiaoguang Gao*^[a]^, Chengzhen Xue^[a]^, Xiaoliang Zhang^[a]^, Xuejuan Meng^[a]^, Xiaochun Li*^[a]^and Li Niu*^[a] [b]^***

*[a] Institute of Biomedical Precision Testing and Instrumentation, College of Artificial Intelligence, Taiyuan University of Technology, Taiyuan, 030024, China*

*[b]* *School of Chemical Engineering and Technology,* *Sun Yat-Sen University, Zhuhai, 519000, China*

E-mail: [gaoxiaoguang@tyut.edu.cn](mailto:gaoxiaoguang@tyut.edu.cn), [lixiaochun@tyut.edu.cn](mailto:lixiaochun@tyut.edu.cn), lniu@gzhu.edu.cn

**The file includes:**

Supplementary Figure S1. Detailed preparation process of the T-P DMES

Supplementary Figure S2. Effects of different kinds of abrasive papers on porous PDMS

Supplementary Figure S3. Effects of ethanol and heating temperature on porous PDMS

Supplementary Figure S4. Effect of the distance between two abrasive papers on porous PDMS

Supplementary Figure S5 Improved hydrophilicity of porous PDMS

Supplementary Figure S6 Micro-CT images of the porous PDMS

Supplementary Figure S7. Optimization of the mass ratio of PEDOT: PSS, Bi_2_Te_3_ and graphene

Supplementary Figure S8. The detection of subtle and intensive human motions

Supplementary Figure S9. Comparison of electronic skins based on quasi-periodic porous structure and random porous structure when used for pressure sensing

Supplementary Figure S10. The decoupling performance of the T-P DMES

Supplementary Figure S11. Comparison of different deep learning models

Supplementary Figure S12. Recognition of different kinds of materials with similar hardness and thermal conductivity

Supplementary Figure S13. Recognition of cotton materials with different textures

Supplementary Figure S14. Recognition of different alloys

Supplementary Table 1. Comparison of the performance of the different electronic skins for material cognition

**Materials and Methods**

**Figure S1. Detailed preparation process of the T-P DMES**

Figure S1 shows the detailed preparation process of the T-P DMES. The abrasive papers no. 2000 was used for the preparation of porous PDMS. After 100 µL of 75 wt % ethanol solution and PDMS are dropped onto the surface of the abrasive paper, the distance between the upper and lower layers of abrasive paper is controlled to form a confined two-dimensional space. The upper and lower abrasive papers are heated to vaporize the ethanol and enter the PDMS. As PDMS solidifies, a porous elastomer with a quasi-periodic porous structure is successfully prepared. The porous PDMS is immersed into the as-prepared PEDOT: PSS/Bi_2_Te_3_/graphene suspensions for 40 min under mild sonication. The interdigital electrodes and copper foil are fixed to output relative resistance change and thermoelectric voltage, respectively.


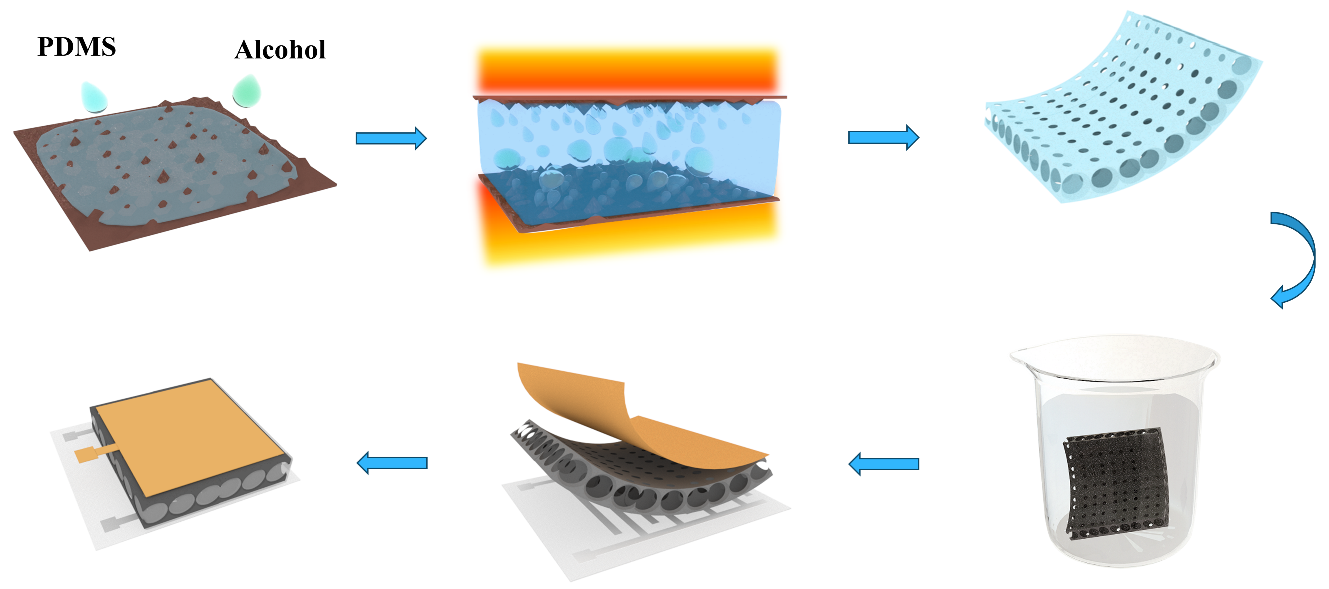


**Figure S1.** The detailed preparation process of the T-P DMES, such as the preparation of porous PDMS, decoration of composites, the fixation of electrodes and the encapsulation of the T-P DMES.

**Figure S2. Effects of different kinds of** **abrasive papers on** **porous PDMS**

According to the classical bubble theory, the size and arrangement of bubbles are greatly affected by their surroundings.^[1,2]^ Therefore, during the preparation of quasi-periodic porous structures, the size and arrangement of the pores are greatly affected by the spinous structure of abrasive paper surface and the confined space formed by the upper and lower layers of abrasive paper. Different kinds of abrasive papers no. 400, 800, 1200, 1500, 2000 and 2500 were used for the preparation of quasi-periodic porous structures. The number of spinous structures per unit area on the abrasive paper surface increases as the number increases, which also means that the larger the number, the smoother the abrasive paper surface. Figure S2a-f show the effect of different kinds of abrasive papers on the preparation of porous PDMS when the concentration of ethanol was 75%. The scale bar in these figures is 1 cm. It can be observed that when abrasive papers no. 400, 800, 1200, 1500 were used to prepare quasi-periodic porous structures, the large spacing between the spinous structures on the abrasive paper results in weak confinement of the bubbles, which leads to a random porous structure. For abrasive paper 2500, its nearly flat surface structure makes it difficult to confine the generated bubbles, so its corresponding porous structure was randomly distributed. It can be observed that the porous structure corresponding to abrasive papers no. 2000 in Figure S2e exhibits a quasi-periodic arrangement. In addition, the size of the porous structure was also relatively consistent. Therefore, abrasive papers no. 2000 was selected for the preparation of porous PDMS.


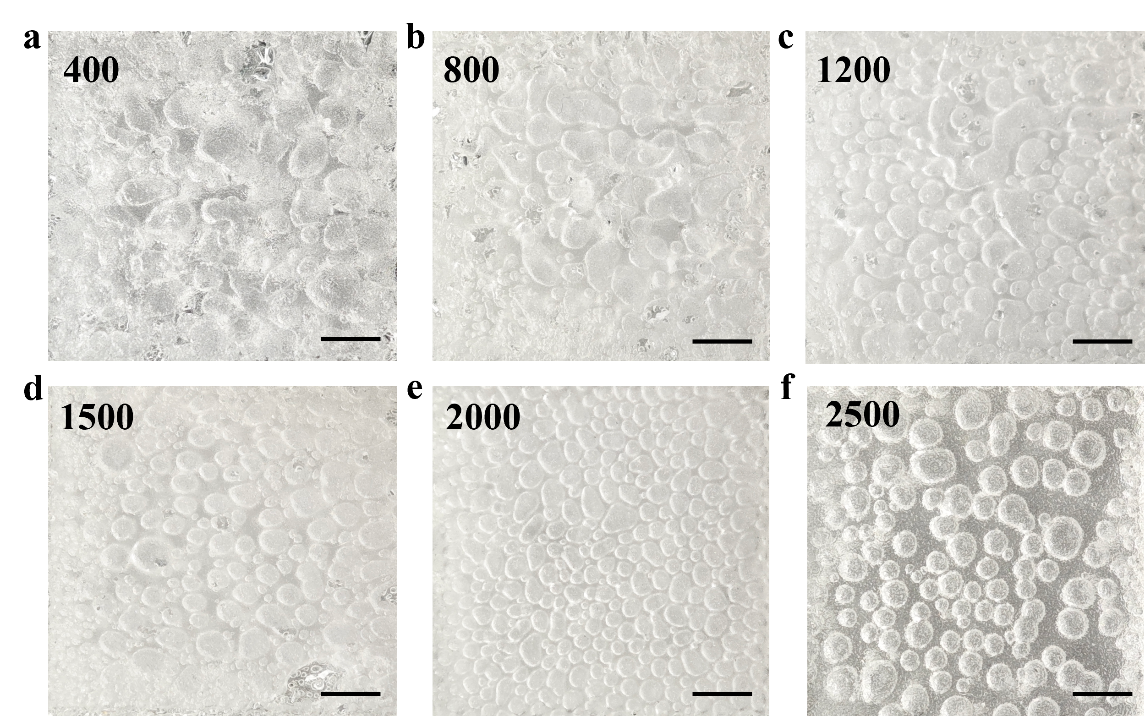


**Figure S2.** The different kinds of abrasive papers no. 400 (a), 800 (b), 1200 (c), 1500 (d), 2000 (e) and 2500 (f) were used for the preparation of porous PDMS.

**Figure S3. Effects of ethanol and heating temperature on porous PDMS**

During the preparation of quasi-periodic porous structures, the concentration of ethanol solution directly affects the generation rate and quantity of bubbles. Ethanol solutions with lower concentrations can only produce a smaller number of bubbles, which ultimately leads to a less porous structure. When the concentrations of the ethanol solution were too high, the rapid bubble generation process will lead to uneven distribution of bubbles in PDMS. Heating temperature also significantly impacts the quasi-periodic porous structure. Lower heating temperatures result in slower ethanol evaporation, preventing the formation of a sufficiently rich porous structure. However, higher heating temperatures can cause ethanol to evaporate instantly, resulting in oversized and unevenly distributed porous structures. Figures S3a and S3b show photographs of porous PDMS prepared with different concentrations of ethanol solutions and different heating temperatures, respectively. It can be observed that the concentration of ethanol solution and the heating temperature have a great impact on the number and diameter of the porous structure of PDMS. The density of porous PDMS prepared with different concentrations of ethanol solutions and different heating temperatures were shown in Figure S3c and S3d. The reduced density of PDMS is due to its rich porous structure, which is very conducive to the decoration of a large amount of composites. Therefore, 75 wt % alcohol solution and heating conditions at 70 °C were used for the preparation of porous PDMS.


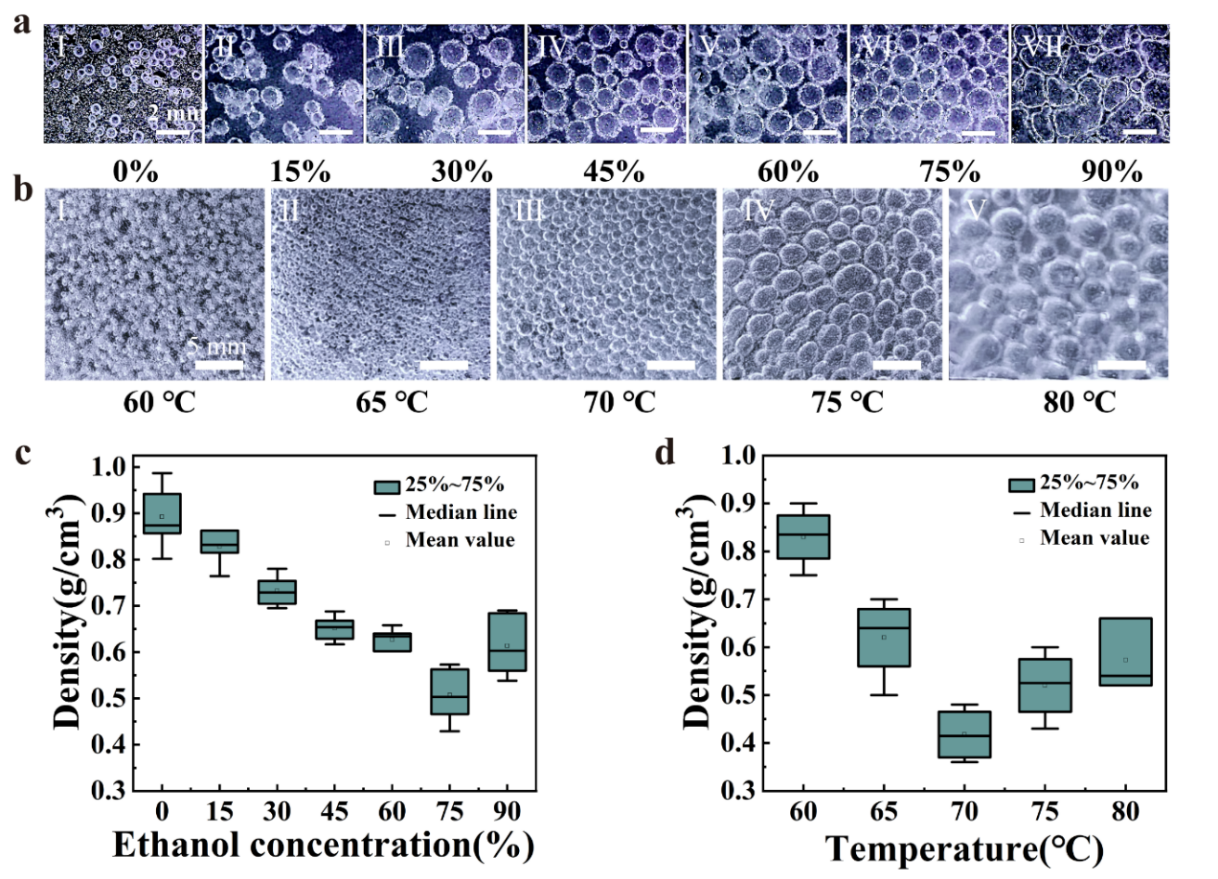


**Figure S3. a-b** Photograph of porous PDMS prepared with different concentrations of ethanol solutions and different heating temperatures. **c-d** The density of porous PDMS prepared with different ethanol concentrations and different heating temperatures.

**Characterizations**

**Figure S4.** **Effect of the distance between two abrasive papers on porous PDMS**

During the preparation of porous PDMS, the upper and lower abrasive papers constitute a confined two-dimensional space. The bubbles generated on the abrasive paper surface interact with PDMS and eventually form a bubble array in a confined two-dimensional space. The photographs of the surface and cross-section of porous PDMS prepared by the upper and lower abrasive papers at different distances are shown in Figure S4a and S4b. It can be observed that when the distance between the abrasive papers varies from 0.5 to 2 mm, the diameter of the porous structure increases with the increase of the distance. In addition, these spherical porous structures show a quasi-periodic arrangement. When the distance between abrasive papers exceeds 2 mm, the porous structure changes from an ordered state to a disordered state. The density and porosity of porous PDMS prepared with different distances of abrasive papers are also provided (Figure S4c and 4d). It can be observed that the density of porous PDMS increases with the increase of the distance between the abrasive papers, while the porosity decreases with the increase of the distance between the abrasive papers. The reduced density of PDMS is due to its rich porous structure, which is very conducive to the decoration of large amounts of composite materials.


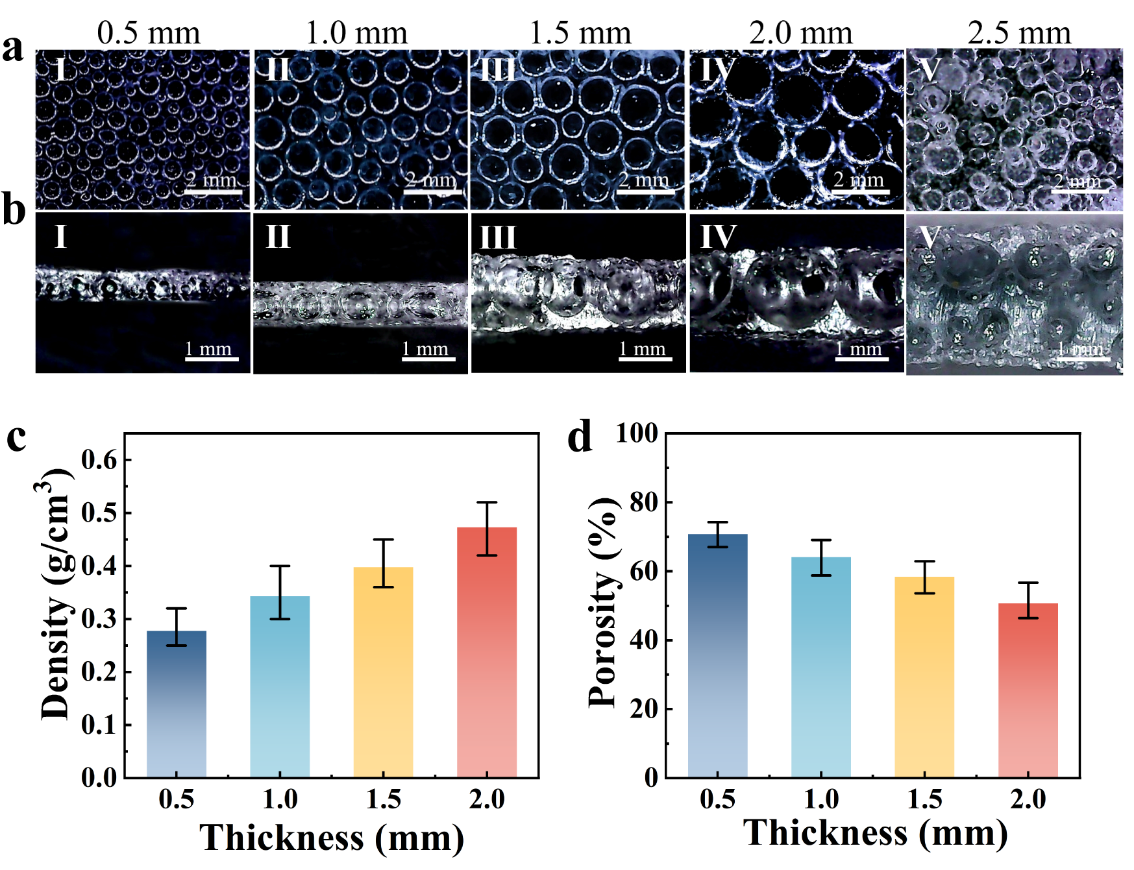


**Figure S4.** **a-b** Photographs of porous PDMS prepared with different distances of abrasive papers. **c-d** Density and porosity of porous PDMS prepared with different distances of abrasive papers.

**Figure S5. Improved hydrophilicity of porous PDMS**

The porous PDMS was treated with UV-ozone to increase its hydrophilicity, which facilitated the successful decoration of graphene, PEDOT: PSS, and Bi_2_Te_3_. Figures S5a and S5b show the contact angles of porous PDMS before and after UV-ozone treatment, respectively. It can be concluded that the hydrophilicity of porous PDMS has been significantly improved from the change of contact angle before (72.9°) and after (53.1°) UV-ozone treatment.


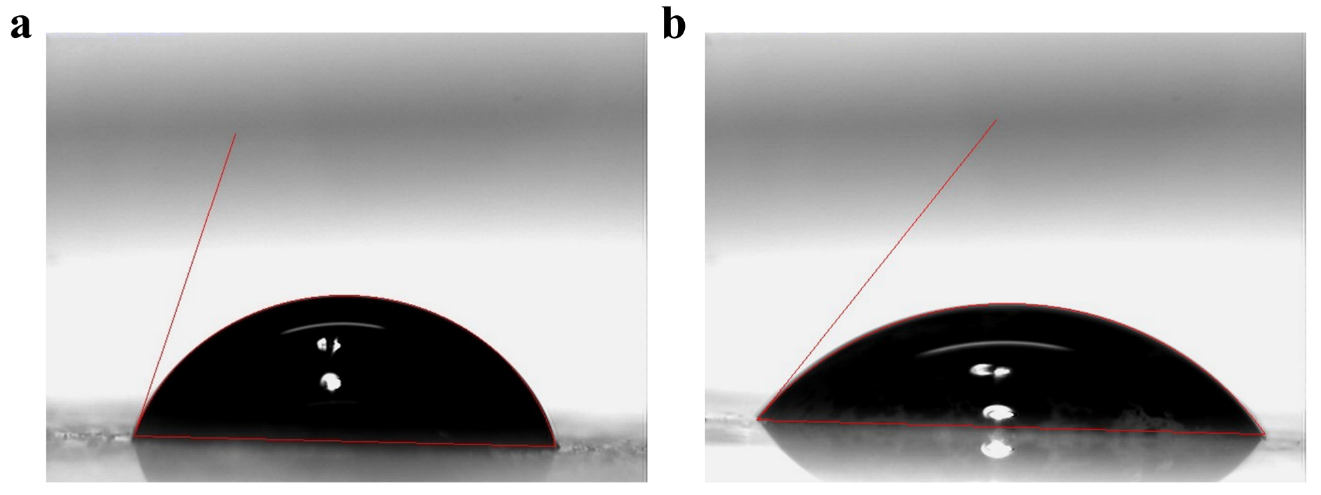


**Figure S5.** Contact angles of porous PDMS before **a** and after UV-ozone treatment **b**.

**Figure S6. Micro-CT images of the porous PDMS**

Due to the presence of the porous structure, X-rays will experience differences in absorption when passing through porous PDMS. Therefore, micro-CT was used to characterize porous PDMS. The CT images of porous PDMS prepared with different distances of abrasive papers are displayed in Figure S6a-b. The scale bars in Figures S6a and b are 1 cm and 2 mm, respectively. The poor contrast in Figure 6a can be observed because the density difference between PDMS and air is not as large as that between bone and air. It can also be observed that the porous structure presents a quasi-periodic arrangement (Figure S6b).

**
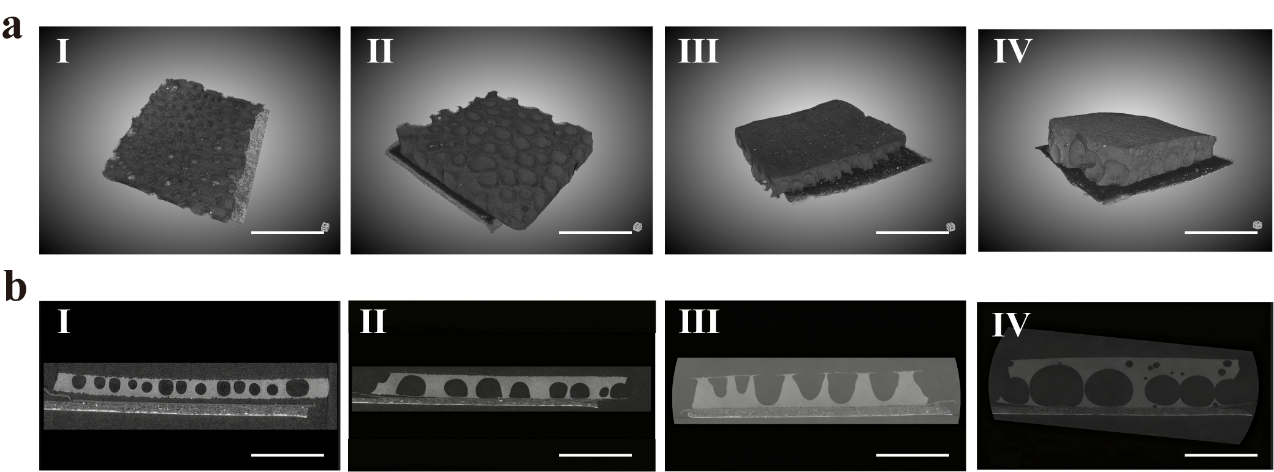
**

**Figure S6.** **a-b** Micro-CT images of porous PDMS prepared with different distances of abrasive papers (0.5 cm, 1.0 cm, 1.5 cm and 2.0 cm).

**Additional experimental results**

**Figure S7.** **Optimization of the mass ratio of PEDOT: PSS, Bi_2_Te_3_ and graphene**

Figure S7a shows the relationship between the thermoelectric voltage output of the T-P DMES and the temperature difference at different PEDOT: PSS and Bi_2_Te_3_ mass ratios. Under different mass ratios, the concentration of PEDOT: PSS in the mixed suspension is 10 mg/mL, and the concentration of Bi_2_Te_3_ is varied. It can be observed that the *S*_T_ reaches maximum when the mass ratio of the PEDOT and Bi_2_Te_3_ is 2:1. A large number of the T-P DMES were prepared to measure *S*_T_ values (Figure S7b). Afterwards, graphene solutions with different concentrations were used to improve the piezoresistive properties of the T-P DMES. It can be observed that the T-P DMES has the largest GF value (50.04 kPa^-1^), when the mass ratio of PEDOT: PSS: Bi_2_Te_3_: graphene is 20:10:1(Figure S7c and 7d).


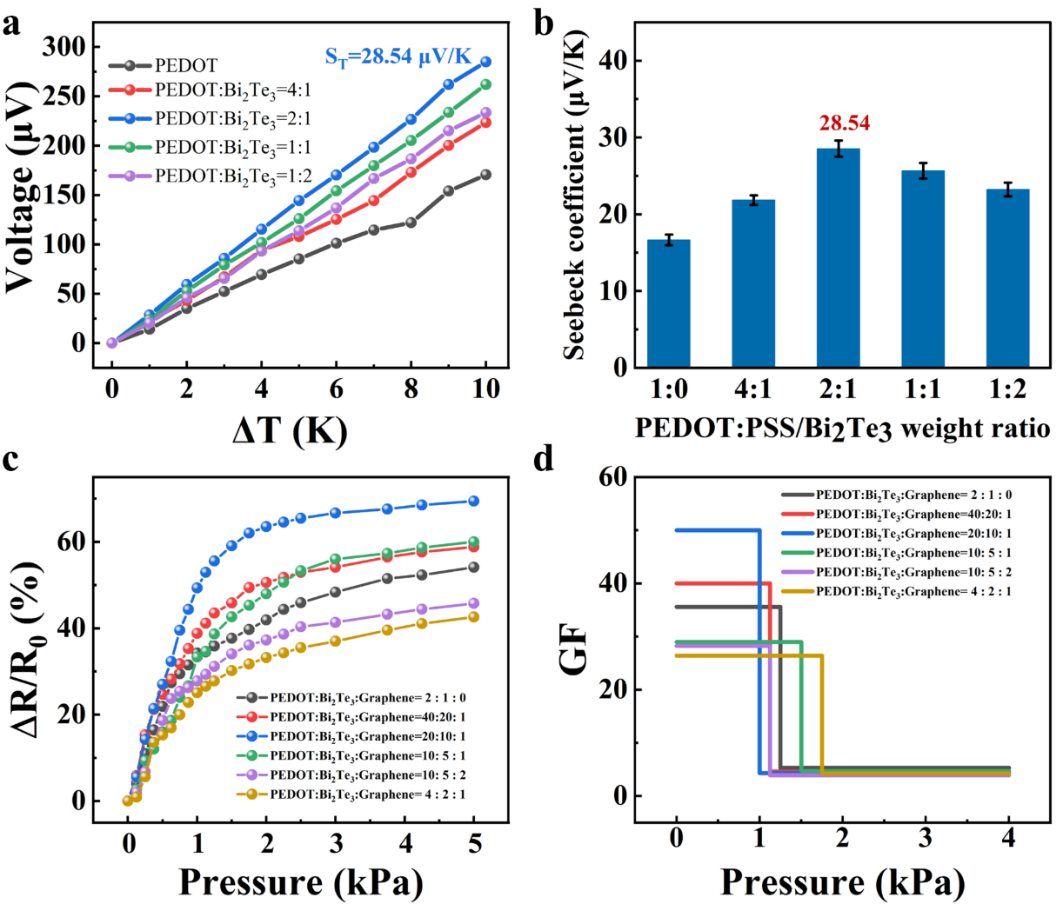


**Figure S7. a-b** Thermoelectric response and *S*_T_ of the T-P DMES at different mass ratios of PEDOT: PSS and Bi_2_Te_3_. **c-d** The piezoresistive properties of the T-P DMES at different mass ratios of PEDOT: PSS, Bi_2_Te_3_ and graphene.

**Figure S8.** **The detection of** **subtle and intensive human motions**

The piezoresistive properties of the T-P DMES are utilized to detect subtle and intensive human motions. In Figures S8a and S8b, subtle human motions such as pulse and blinking can cause obvious changes in the relative resistance of the T-P DMES. In addition, the wider detection range of the T-P DMES also enables the detection of intensive human motions such as wrist bending (Figures S8c), finger bending (Figures S8d), arm bending (Figures S8e), and knee bending (Figures S8f).

**
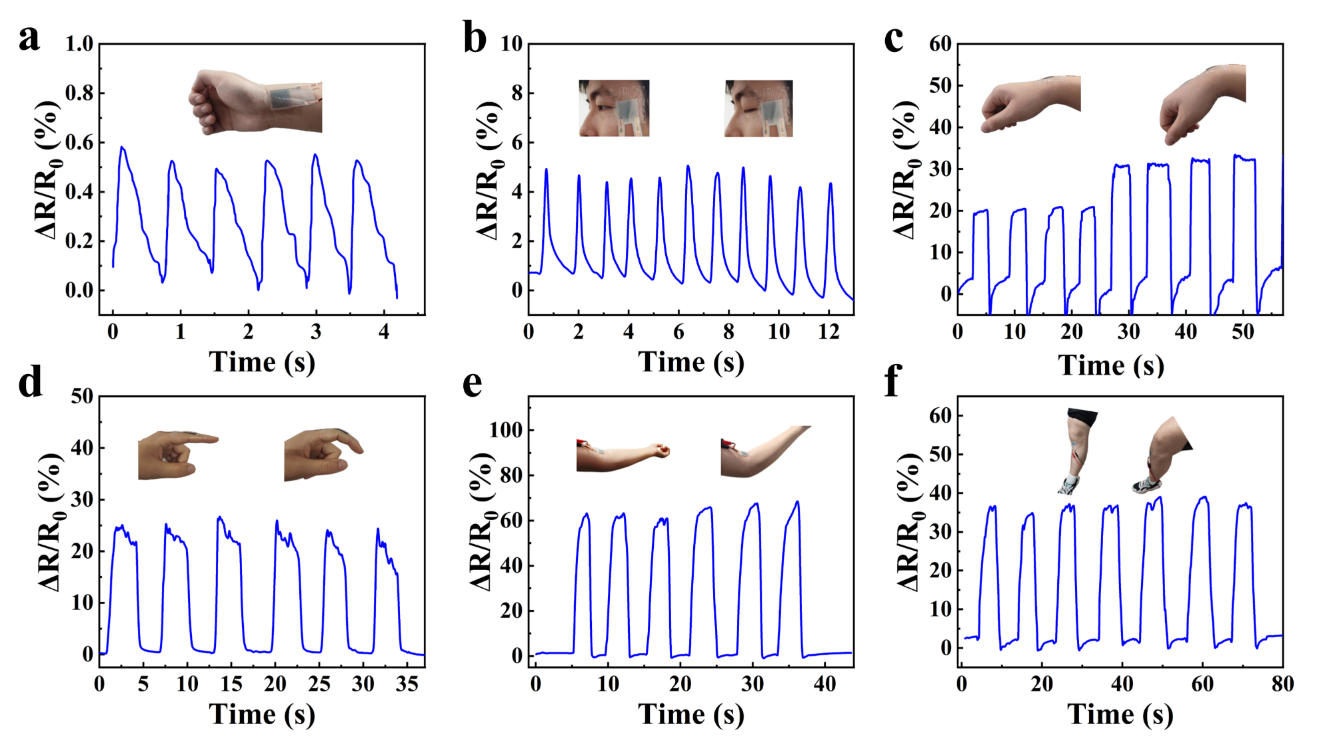
**

**Figure S8. a-b** The relative resistance changes of the T-P DMES in response to subtle human movements such as pulse and blinking. **c-f** The relative resistance changes of the T-P DMES in response to intensive human motions such as wrist bending, finger bending, arm bending, and knee bending.

**Figure S9.** **Comparison of** **electronic skins based on quasi-periodic porous structure and random porous structure when used for pressure sensing**

The pressure performance of the electronic skin based on the quasi-periodic porous structure we proposed was compared with that of the electronic skin based on the random porous structure. The electronic skin based on the random porous structure was prepared using sugar cube as a template. In addition, the mass ratios of Bi_2_Te_3_, PEDOT:PSS, and graphene used in two kinds of electronic skins are the same. It can be observed that the electronic skin we proposed has a significant advantage in terms of sensitivity (Figure S9a and 9b). To investigate the uniformity of the electronic skin, a 0.5 cm diameter weight was placed on the surface of the electronic skins and moved to acquire the signal response of the electronic skins. During the scanning process, the weight was moved in steps of 1 mm. The real-time response of the electronic skins, along with the signal corresponding to each pixel during the movement of the weight was recorded, as displayed in Figures S9c and 9d. It can be observed that the signal of the electronic skin based on the quasi-periodic porous structure is basically consistent during the movement of the weight, while the signal of the electronic skin based on the random porous structure fluctuates significantly. Therefore, the electronic skin based on quasi-periodic porous structures exhibits significant advantages in pressure sensing.

**
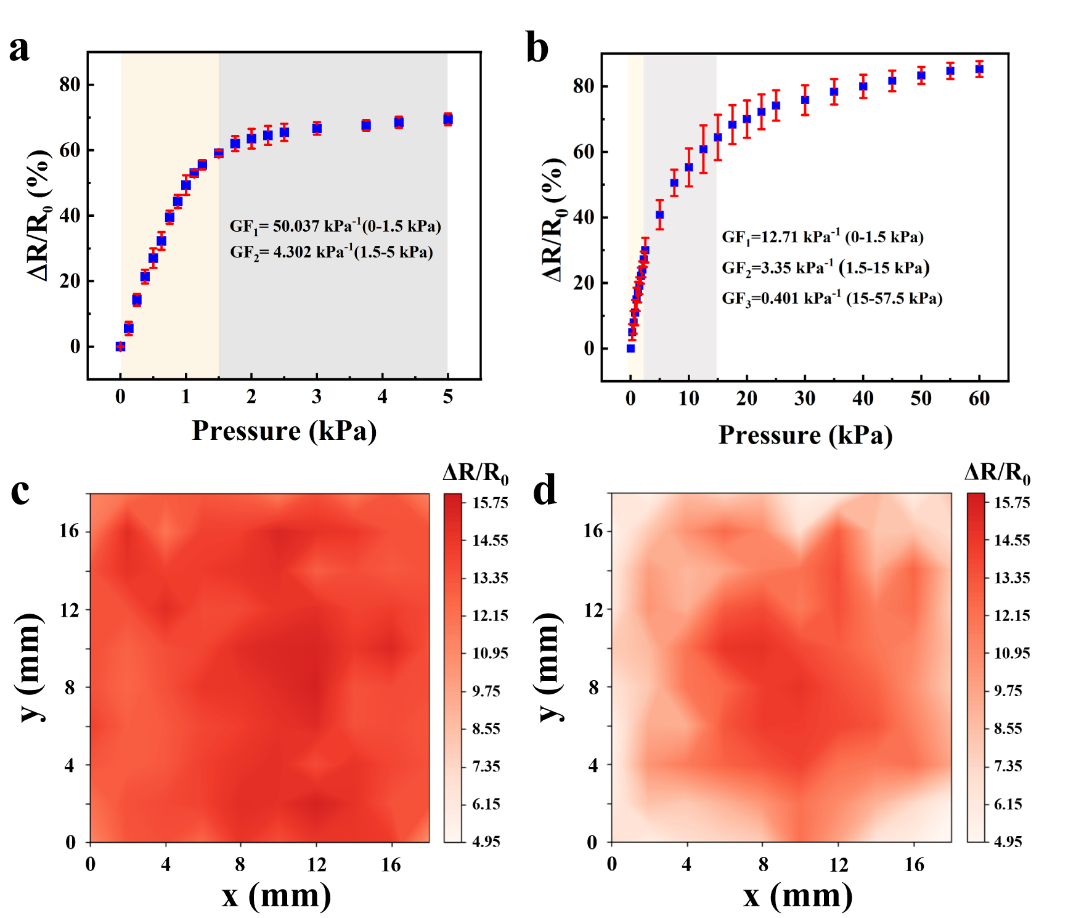
**

**Figure S9.** a-b Changes in relative resistance as a function of pressure corresponding to electronic skins based on quasi-periodic porous structure and random porous structure. c-d Scanned responses of the electronic skins under the movement of the weight.

**Figure S10.** **The decoupling performance of the T-P DMES**

The different application scenarios are used to verify the decoupling performance of the T-P DMES. In Figure S10a, a beaker filled with room temperature water is placed on the surface of the T-P DMES. It can be observed that the relative resistance of the T-P DMES changes significantly due to the pressure loaded by the beaker. However, the output thermoelectric voltage is zero considering there is no temperature difference between the beaker and the T-P DMES. In Figure S10b, a beaker filled with hot water is placed on the surface of the T-P DMES. It can be observed that not only the relative resistance change caused by the pressure loaded by beaker, but also the thermoelectric voltage caused by the temperature difference between the beaker and the T-P DMES. Figure S10c shows the signal changes as a beaker filled with hot water approaches and moves away from the T-P DMES. It can be observed that the relative resistance of the T-P DMES did not change due to the absence of pressure loaded by the beaker. However, a temperature difference between the upper and lower surfaces of the T-P DMES will be generated when the beaker approaches the T-P DMES, eventually generating a significant thermoelectric voltage. The above experimental results all demonstrated that the T-P DMES can respond to temperature and pressure stimuli without crosstalk.

**
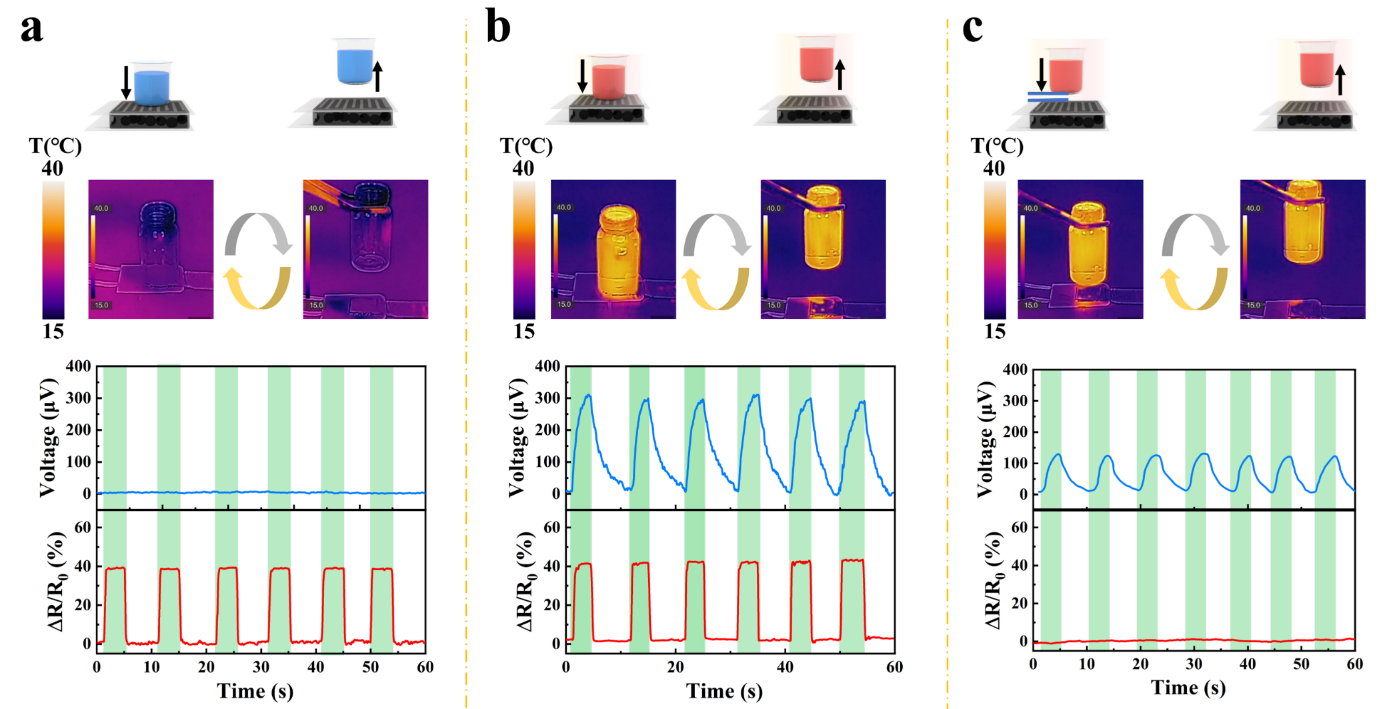
**

**Figure S10.** **a-b** Signal response caused by beakers containing room temperature water and hot water in contact with the T-P DMES. **c** The signal response caused by a beaker approaching and moving away from the T-P DMES.

**Figure S11.** **Comparison of different deep learning models**

In this study, the T-P DMES was combined with CNN model for material recognition. Its performance was compared with other deep learning models such as Lenet-5, VGG16, ResNet and AlexNet. Figures S11a-d show the comparison of accuracy and loss value of these deep learning models. It can be observed that compared with other deep learning models, the CNN model shows superior performance in the field of material recognition.


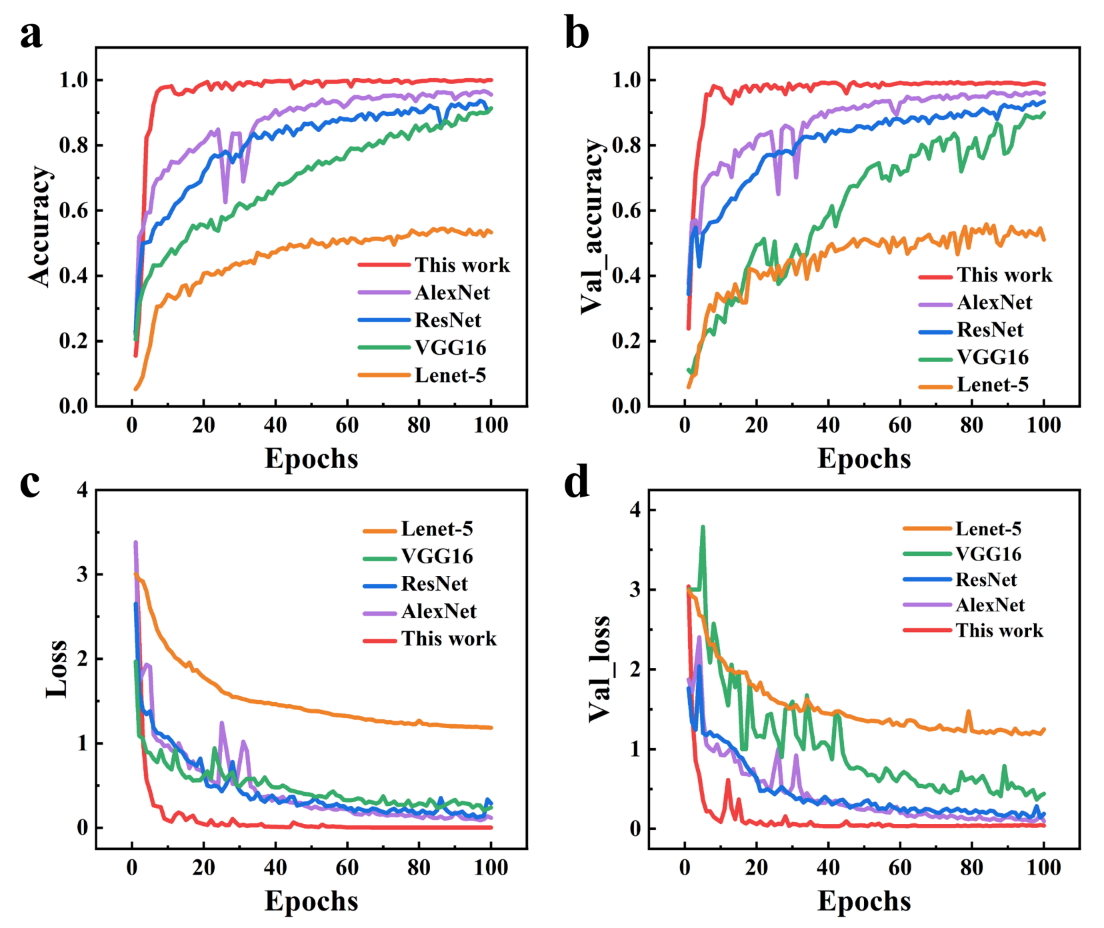


**Figure S11. a-c** Diagrams of identification accuracy and loss function of two data sets. **d** The comparison of accuracy, loss value, and response time of these deep learning models.

**Figure S12.** **Recognition of different kinds of materials with similar hardness and thermal conductivity**

Figure S12a shows photographs of 13 different materials with similar hardness or thermal conductivity. During the contact between the T-P DMESs and different kinds materials, the travel the relative resistance change and thermoelectric voltage are recorded. Figure S12b and S12c show the distinction cluster of the relative resistance change and thermoelectric voltage datasets collected from 13 different materials, respectively. Figure S12d shows the distinction cluster of the mixed dataset collected from 13 different materials. It can be observed that mixed data set consisting of relative resistance changes and thermoelectric voltages can be well visualized and distinguished in 2D space. The output confusion matrices confirmed material cognition with accuracies of 87.3% (Figure S12e) and 85.0% (Figure S12f) for the relative resistance change and thermoelectric voltage datasets, respectively. For the mixed dataset, the accuracy rate reaches 98.7% (Figure S12g).


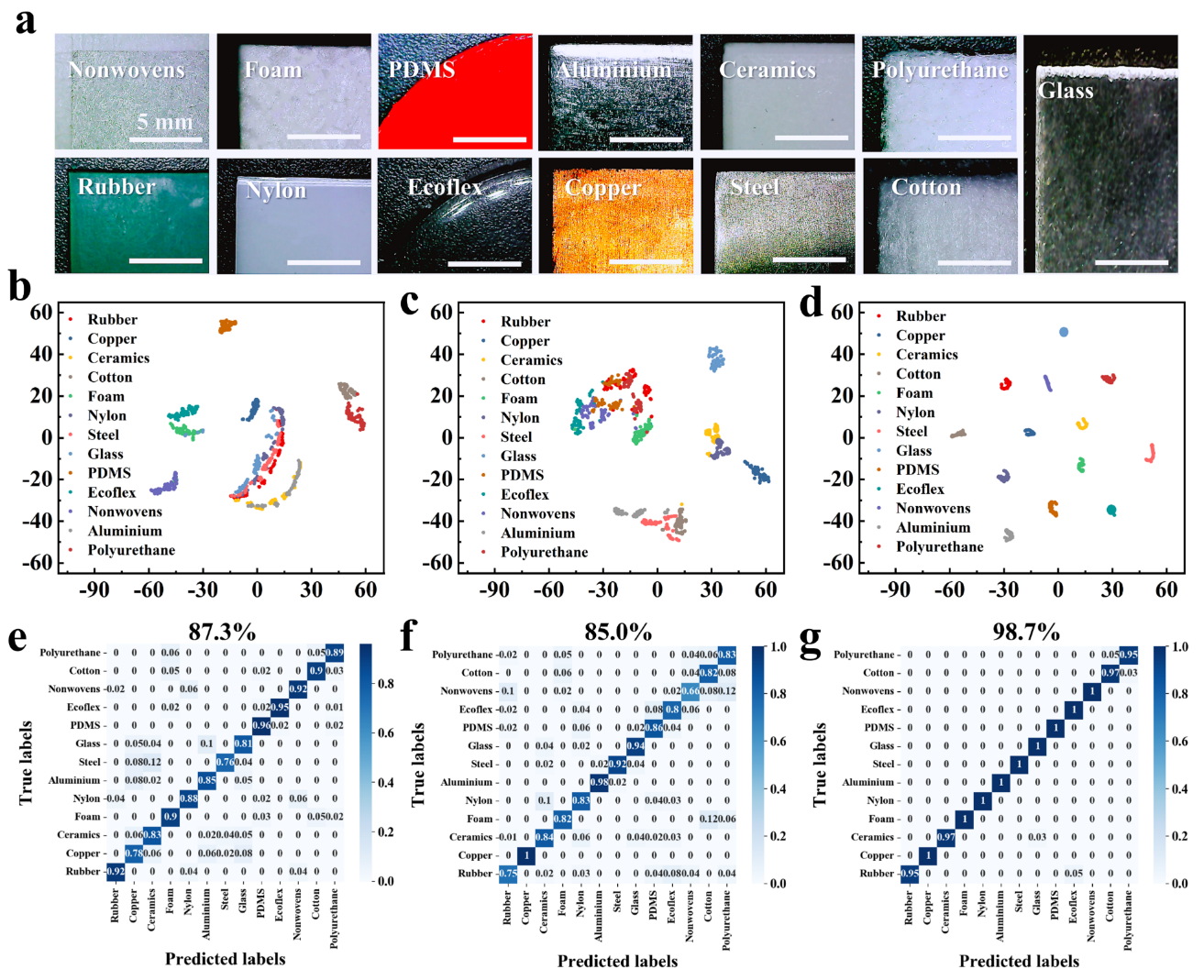


**Figure S12. a** Photographs of 13 selected materials with similar hardness or thermal conductivity. **b-d** T-SNE visualization of the data set collected from the 13 selected materials. **e-g** Confusion matrix for material cognition using the relative resistance change dataset, the thermoelectric voltage dataset, and the mixed dataset.

**Figure S13.** **Recognition of cotton materials with different textures**

Figure S13a shows photographs of 12 cotton materials with different textures. Figures S13b and S13c show the distinction cluster of the relative resistance change and thermoelectric voltage datasets collected from 12 different materials, respectively. Figure S13d shows the distinction cluster of the mixed dataset collected from 12 different materials. It can be observed that the mixed data set can be well visualized and distinguished in 2D space. The output confusion matrices confirmed material cognition with accuracies of 93.9% (Figure S13e) and 82.9% (Figure S13f) for the relative resistance change and thermoelectric voltage datasets, respectively. For the mixed dataset, the accuracy rate reaches 99.1% (Figure S13g).

**
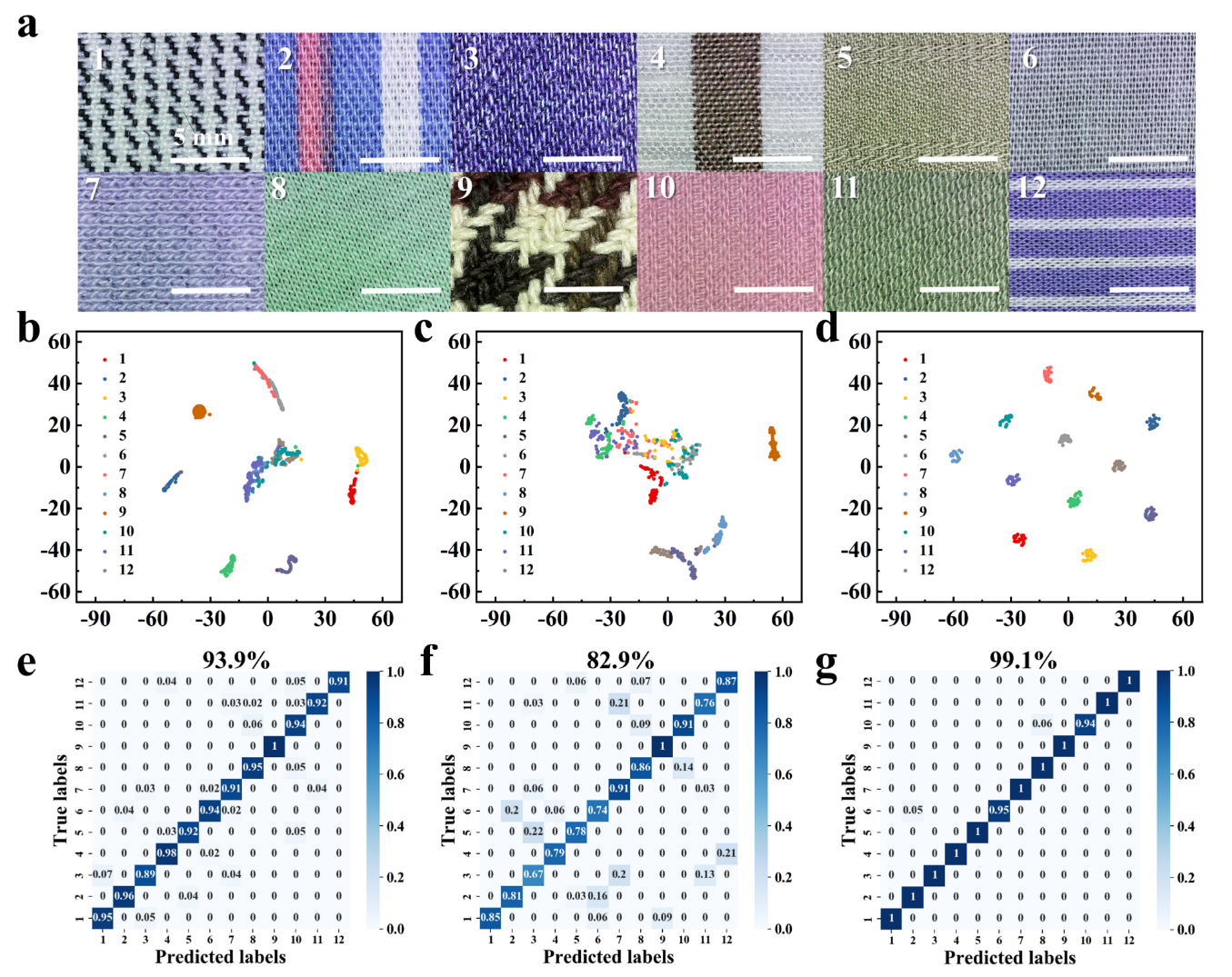
**

**Figure S13.** **a** Photographs of 12 selected cotton materials with different textures. **b-d** T-SNE visualization of the data set collected from the 12 selected materials. **e-g** Confusion matrix for material cognition using the relative resistance change dataset, the thermoelectric voltage dataset, and the mixed dataset.

**Figure S14.** **Recognition of different alloys**

Figure S14a shows photographs of four different steel alloys (No. 201, 304, 316 and 430) and four different aluminum alloys (No. 1060, 5052, 6061 and 7075). Figures S14b and S14c show the distinction cluster of the relative resistance change and thermoelectric voltage datasets collected from these different alloys, respectively. Figure S14d shows the distinction cluster of the mixed dataset. It can be observed that the mixed data set can be well visualized and distinguished in 2D space. The output confusion matrices confirmed material cognition with accuracies of 86.4% (Figure S14e) and 80.3% (Figure S14f) for the relative resistance change and thermoelectric voltage datasets, respectively. For the mixed dataset, the accuracy rate reaches 95.1% (Figure S14g).


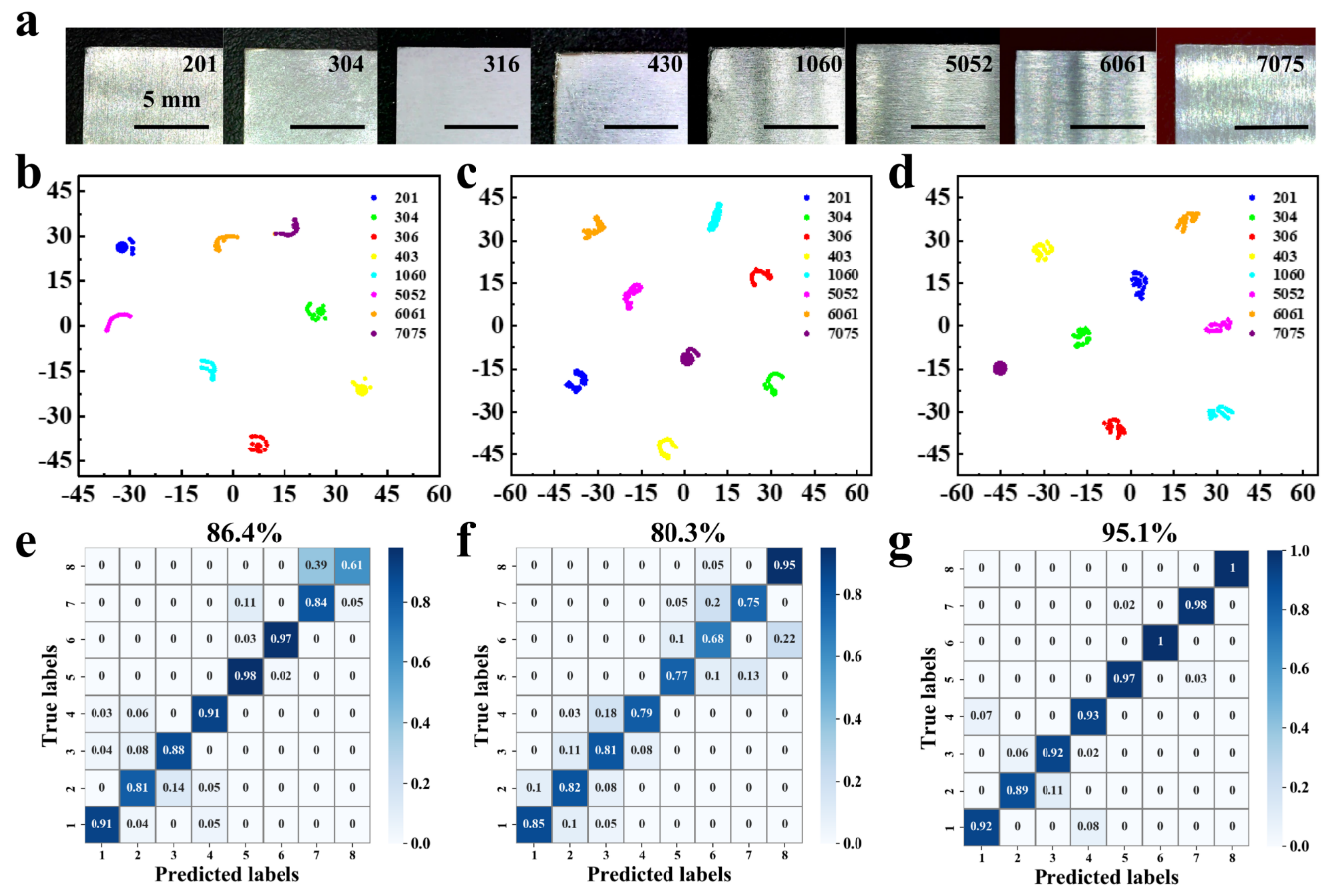


**Figure S14. a** Photographs of 8 selected alloys. **b-d** T-SNE visualization of the data set collected from the 8 selected alloys. **e-g** Confusion matrix for material cognition using the relative resistance change dataset, the thermoelectric voltage dataset, and the mixed dataset.

**Table S1. Comparison of the performance of the different electronic skins for material cognition**

| Electronic skin | | Material cognition | | | |
| --- | --- | --- | --- | --- | --- |
| Working mechanism | Modal | Material Type | Principle | Accuracy | References |
| Piezoresistive-Thermoelectric | Dual | 33 | Hardness, Thermal conductivity | 97.64% | This work |
| Capacitive-Triboelectric | Dual | 12 | Charge property | 98.34% | ^[3]^ |
| Capacitive | Single | 8 | Hardness | - | ^[4]^ |
| Capacitive | Single | 20 | Texture | 98.90% | ^[5]^ |
| Triboelectric | Single | 7 | Charge property | 96.80% | ^[6]^ |
| Piezoresistive-Triboelectric | Dual | 12 | Texture, Charge property | 99.10% | ^[7]^ |
| Piezoresistive | Single | 4 | Hardness | - | ^[8]^ |
| Piezoresistive-Triboelectric | Single | 11 | Charge property | 94.44% | ^[9]^ |
| Capacitive-Triboelectric | Single | 5 | Charge property | - | ^[10]^ |
| Piezoresistive | Single | 6 | Texture | 91.40% | ^[11]^ |
| Piezoresistive | Single | 4 | Texture | - | ^[12]^ |

**Supplementary References**

[1] Z. Huang, M. Su, Q. Yang, Z. Li, S. Chen, Y. Li, X. Zhou, F. Li, Y. Song, A general patterning approach by manipulating the evolution of two-dimensional liquid foams. *Nat. Commun.* **2017**, *8* (1), 14110.

[2] J. Bae, K. Lee, S. Seo, J. G. Park, Q. Zhou, T. Kim, Controlled open-cell two-dimensional liquid foam generation for micro-and nanoscale patterning of materials *Nat. Commun.* **2019**, *10* (1), 3209.

[3] H. Niu, H. Li, S. Gao, Y. Li, X. Wei, Y. Chen, W. Yue, W. Zhou, G. Shen, Perception-to-Cognition Tactile Sensing Based on Artificial-Intelligence-Motivated Human Full-Skin Bionic Electronic Skin. *Adv. Mater.* **2022**, *34* (31), 2202622.

[4] C. Lv, C. Tian, J. Jiang, Y. Dang, Y. Liu, X. Duan, Q. Li, X. Chen, M. Xie, Ultrasensitive Linear Capacitive Pressure Sensor with Wrinkled Microstructures for Tactile Perception. *Adv. Sci.* **2023**, *10* (14), 2206807.

[5] N. Bai, Y. Xue, S. Chen, L. Shi, J. Shi, Y. Zhang, X. Hou, Y. Cheng, K. Huang, W. Wang, A robotic sensory system with high spatiotemporal resolution for texture recognition. *Nat. Commun.* **2023**, *14* (1), 7121.

[6] X. Qu, Z. Liu, P. Tan, C. Wang, Y. Liu, H. Feng, D. Luo, Z. Li, Z. L. Wang, Artificial tactile perception smart finger for material identification based on triboelectric sensing *Sci. Adv.* **2022**, *8* (31), eabq2521.

[7] S. Chun, W. Son, H. Kim, S. K. Lim, C. Pang, C. Choi, Self-powered pressure-and vibration-sensitive tactile sensors for learning technique-based neural finger skin. *Nano Lett.* **2019**, *19* (5), 3305.

[8] L. Beker, N. Matsuhisa, I. You, S. R. A. Ruth, S. Niu, A. Foudeh, J. B.-H. Tok, X. Chen, Z. Bao, A bioinspired stretchable membrane-based compliance sensor. *PNAS.* **2020**, *117* (21), 11314.

[9] Y. Pang, X. Xu, S. Chen, Y. Fang, X. Shi, Y. Deng, Z.-L. Wang, C. Cao, Skin-inspired textile-based tactile sensors enable multifunctional sensing of wearables and soft robots. *Nano Energy* **2022**, *96*, 107137.

[10] S.-R. Kim, S. Lee, J.-W. Park, A skin-inspired, self-powered tactile sensor *Nano Energy* **2022**, *101*, 107608.

[11] Y. Guo, X. Wei, S. Gao, W. Yue, Y. Li, G. Shen, Recent advances in carbon material‐based multifunctional sensors and their applications in electronic skin systems *Adv. Funct. Mater.* **2021**, *31* (40), 2104288.

[12] D. Lu, T. Liu, X. Meng, B. Luo, J. Yuan, Y. Liu, S. Zhang, C. Cai, C. Gao, J. Wang, Wearable triboelectric visual sensors for tactile perception. *Adv. Mater.* **2023**, *35* (7), 2209117.
